# The PPARα Agonist Fenofibrate Prevents Formation of Protein Aggregates (Mallory-Denk bodies) in a Murine Model of Steatohepatitis-like Hepatotoxicity

**DOI:** 10.1038/s41598-018-31389-3

**Published:** 2018-08-28

**Authors:** Aniket Nikam, Jay V. Patankar, Meghana Somlapura, Pooja Lahiri, Vinay Sachdev, Dagmar Kratky, Helmut Denk, Kurt Zatloukal, Peter M. Abuja

**Affiliations:** 1 Institute of Pathology, Medical University of Graz, Graz, Austria; 2 Gottfried Schatz Research Centre, Medical University of Graz, Graz, Austria; 3 Present Address: University of Miami, Miller School of Medicine, Department of Surgery, Miami, Florida USA; 4 Present Address: Department of Medicine 1, Friedrich-Alexander-University, D-91054 Erlangen, Germany

## Abstract

Chronic intoxication of mice with the porphyrinogenic compound 3,5-diethoxycarbonyl-1,4-dihydrocollidine (DDC) leads to morphological and metabolic changes closely resembling steatohepatitis, a severe form of metabolic liver disease in humans. Since human steatohepatitis (both the alcoholic and non-alcoholic type) is characterized by reduced expression of PPARα and disturbed lipid metabolism we investigated the role of this ligand-activated receptor in the development of DDC-induced liver injury. Acute DDC-intoxication was accompanied by early significant downregulation of *Pparα* mRNA expression along with PPARα-controlled stress-response and lipid metabolism genes that persisted in the chronic stage. Administration of the specific PPARα agonist fenofibrate together with DDC prevented the downregulation of PPARα-associated genes and also improved the stress response of Nrf2-dependent redox-regulating genes. Moreover, oxidative stress and inflammation were strongly reduced by DDC/fenofibrate co-treatment. In addition, fenofibrate prevented the disruption of hepatocyte intermediate filament cytoskeleton and the formation of Mallory-Denk bodies at late stages of DDC intoxication. Our findings show that, like in human steatohepatitis, PPARα is downregulated in the DDC model of steatohepatitis-like hepatocellular damage. Its downregulation and the pathomorphologic features of steatohepatitis are prevented by co-administration of fenofibrate.

## Introduction

Non-alcoholic steatohepatitis (NASH) is a severe form of metabolic liver disease that can progress to cirrhosis and hepatocellular carcinoma^1^. It is assumed to evolve from simple steatosis, a benign manifestation of non-alcoholic (and also alcoholic) liver disease as a consequence of deregulation of hepatic lipid metabolism. The progression might be due to additional ‘hits’^2^, possibly related to oxidative stress. Consequentially, metabolic deregulation, in particular alterations of lipid and energy metabolism, and the role of oxidative stress have become a focus in the investigation of the pathogenesis of NASH.

Peroxisome proliferator-activated receptor α (PPARα) is a transcription factor that regulates transcription by binding to PPARα response elements of a broad variety of genes^3^, among them key genes involved in mitochondrial and peroxisomal β-oxidation, as well as in stress response and inflammation^3^. Recent studies have shown that PPARα expression is significantly reduced in livers of patients with NASH but not with simple steatosis^4–6^. PPARα agonists, specifically fibrates, have been previously suggested also as treatment for fatty liver disease, but appeared problematic due to side effects^7^. Recently, however, a combined PPARα/δ agonist showed promising therapeutic effects^8^.

Since the roles of PPARα in the liver of humans and mice are similar^9^, mouse models that display the morphological NASH phenotype are useful in elucidating the role of PPARα. However, experimental deregulation of lipid metabolism alone, e.g. by feeding mice high-fat diet or a methionine-choline deficient diet, does not reproduce the full morphologic spectrum of (human) NASH^10^. Hence, at least a second insult, such as mitochondrial dysfunction, must be postulated as trigger.

We have previously described mitochondrial dysfunction in a mouse model that generates the phenotypic characteristics of NASH, such as cytoplasmic protein inclusions termed Mallory-Denk bodies (MDBs), steatosis, hepatocyte ballooning, disruption of the keratin intermediate filament cytoskeleton, inflammation, and apoptosis^11^. While mitochondrial dysfunction develops already early, after about 2 weeks, the morphological characteristics of NASH develop only after prolonged intoxication (8–10 weeks) of mice with the porphyrinogenic compound 3,5-diethoxycarbonyl-1,4-dihydrocollidine (DDC). Since the primary pathogenic mechanism of DDC intoxication is the inhibition of ferrochelatase, the last step in heme biosynthesis^12,13^, the impaired synthesis of mitochondrial heme proteins might be responsible for - or at least contribute to - mitochondrial dysfunction. In line with this assumption, ferrochelatase-deficient (*Fech*^*−/−*^) mice produce MDBs spontaneously at older age and show alterations of ATP, keratins and transglutaminase 2 similar to DDC-intoxicated mice; however, this process takes considerably longer than the evolution of the phenotype in the DDC model, which again suggests the involvement of additional factors. This is further supported by the observation that the ferrochelatase-deficiency-dependent lesions were aggravated by DDC administration^14^.

In the past, chronically DDC-intoxicated mice have been useful to study alterations of the keratin intermediate filament cytoskeleton in hepatocytes and their association with MDBs^15,16^. These mainly consist of aggregated misfolded/partially degraded keratins, heat shock proteins, ubiquitin and the adapter protein sequestosome 1/p62 (p62) and are morphologic key features of human alcoholic steatohepatitis (ASH) and NASH. Both types of steatohepatitis in humans are associated with metabolic alterations leading to hepatic steatosis, oxidative stress, and mitochondrial dysfunction with impaired energy metabolism^17–19^, which were also found in rodent models^10,11,20,21^. Notably, while DDC-intoxicated mice show many hallmarks of NASH, fat accumulation in this model is rather low and occurs late, in the fully developed stage, suggesting that while steatosis is one possible precursor of NASH it is not the only factor that can predispose to NASH.

The development of NASH involves deregulation of various metabolic processes, several of which are regulated by PPARα^6^, such as energy metabolism and a variety of other metabolic functions. Here we investigated the role of PPARα in the DDC model^11,22^ and observed reduced expression already during early stages of intoxication, preceding the appearance of the NASH-like phenotype. This was accompanied by downregulation of PPARα-dependent genes involved in lipid metabolism, and, interestingly, also by impaired stress response. To confirm that the observed effects are indeed related to PPARα downregulation we used the PPARα-specific agonist fenofibrate to restore the expression of PPARα - dependent genes.

## Results

### DDC treatment rapidly decreased the expression of *Pparα* and PPARα-dependent genes of fatty acid metabolism and stress response in mouse liver

**Figure 1:**
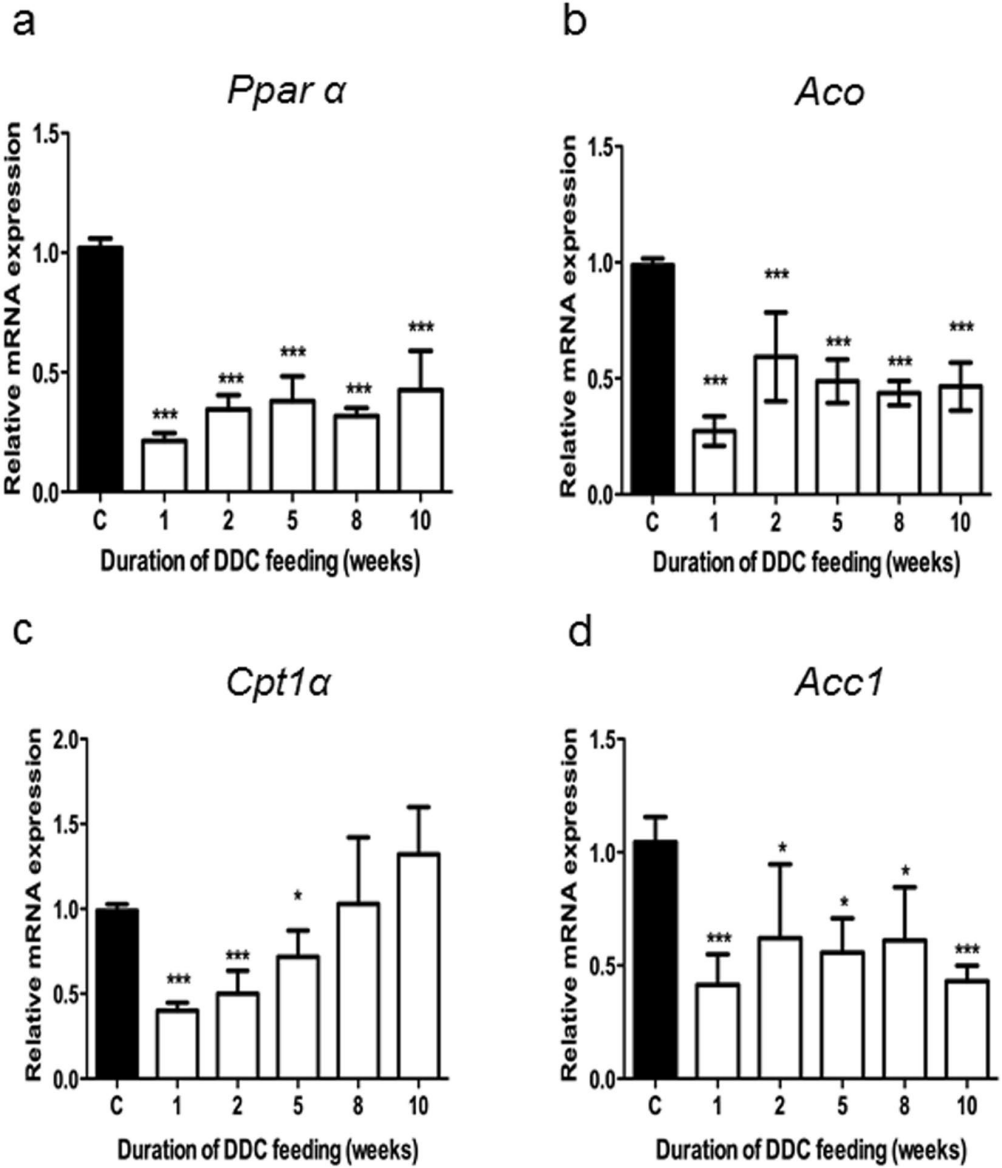
DDC intoxication leads to downregulation of *Pparα* and lipid metabolism-related genes. Normalized expression levels of (**a**) *Pparα*; (**b**) acyl-CoA oxidase (*Aco)*; (**c**) carnitine-palmitoyl transferase 1α (*Cpt1α*); (**d**) acyl-CoA carboxylase 1 (*Acc1)* were evaluated by qRT-PCR of mRNA extracted from whole liver tissue. Data are represented as mean ± SD collected from 5 animals per treatment group and shown as fold-change relative to controls. Significances of DDC intoxication stages are shown versus control: *p < 0.05; ***p < 0.001.

Already after 1 week of DDC treatment, *Pparα* expression was about fourfold downregulated as revealed by qRT-PCR (Fig. 1a) and Western blotting (see Supplementary Fig. SF1). It remained significantly, i.e. 2-3-fold, downregulated for the whole duration of the experiment, until 10 weeks of DDC intoxication (Fig. 1a). The mRNA-expression of several PPARα-dependent genes, such as peroxisomal acyl-CoA carboxylase 1 (*Acc1*), mitochondrial acyl-CoA-oxidase (*Aco*), and carnitine-palmitoyl-transferase 1α (*Cpt1α*) which play important roles in the liver regarding energy, cholesterol and fatty acid metabolism, decreased accordingly (Fig. 1b–d). Interestingly, *Cpt1α* expression normalized at later stages (8–10 weeks) of DDC treatment despite low *Pparα* expression while *Acc1* and *Aco* expression remained reduced.

**Figure 2:**
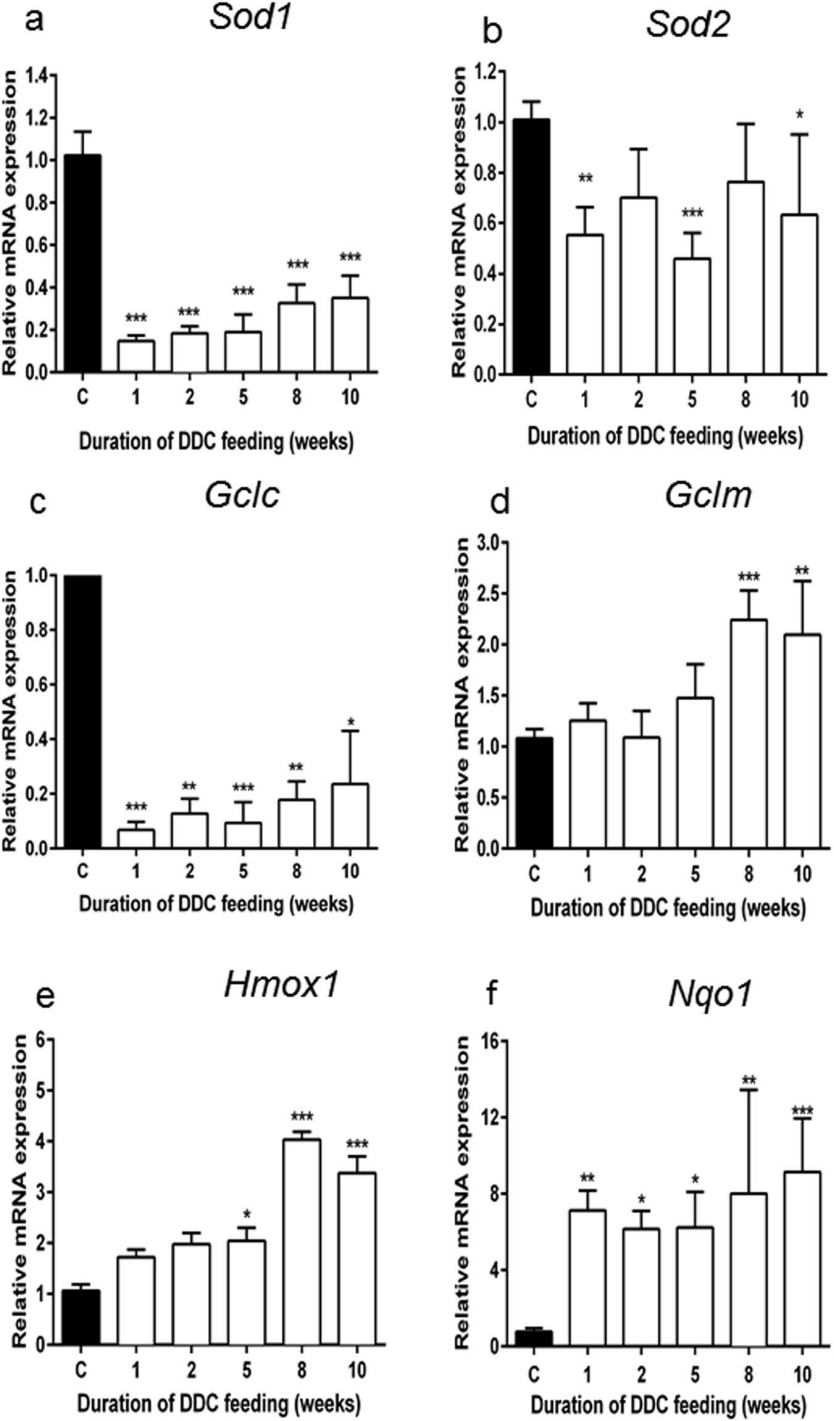
Effect of DDC on the expression of antioxidant and stress-response genes. Normalized expression levels of (**a**) superoxide dismutase 1 (*Sod1)*, (**b**) superoxide dismutase 2 (*Sod2)*, (**c**) glutamate-cysteine ligase, catalytic subunit (*Gclc)*, (**d**) glutamate-cysteine ligase, modifier subunit (*Gclm)*, (**e**) heme oxygenase 1 (*Hmox1)*, and (**f**) NAD(P)H dehydrogenase quinone 1 (*Nqo1)* were evaluated by qRT-PCR of mRNA extracted from whole liver tissue. Data are represented as mean ± SD collected from 5 animals per treatment group and shown as fold-change relative to controls. Significances of DDC intoxication stages are shown versus controls: *p < 0.05; **p < 0.01; ***p < 0.001.

In addition, we observed rapid and strong downregulation of antioxidant and stress response genes known to be controlled by nuclear factor (erythroid-derived 2)-like 2 (Nrf2), such as Cu-Zn-superoxide dismutase and – to a lesser and more variable extent - Mn- superoxide dismutase (*Sod1* and *Sod2*, respectively), and γ-glutamyl-cysteinyl ligase (*Gclc*), the regulatory subunit of the rate-limiting enzyme of glutathione biosynthesis (Fig. 2a–c). Interestingly, DDC treatment at early stages did not affect the expression of other stress response genes like *Hmox1*, heme oxygenase 1, and *Gclm*, the regulatory subunit of γ-glutamyl-cysteinyl ligase, whereas in the later stages their expression was significantly enhanced. Expression of NAD(P)H-quinone oxidoreductase 1 (*Nqo1*), involved in superoxide scavenging and prevention of one-electron reduction of quinones, was significantly increased in all stages of intoxication (Fig. 2d–f).

### Effect of DDC/fenofibrate co-treatment on parameters of liver damage

Next we studied mice that were simultaneously fed DDC and the PPARα agonist fenofibrate for short (1 and 2 weeks) and long periods (10 weeks). The short-term regimen was chosen because *Pparα* and dependent genes became downregulated within one week, the long-term regimen because PPARα remained downregulated until this time and the NASH-like chronic intoxication phenotype described below in detail appeared after 8–10 weeks. Fenofibrate was selected because it is an effective PPARα agonist and co-activation of other PPAR isotypes, in particular PPARγ, is negligible^23^.

**Figure 3:**
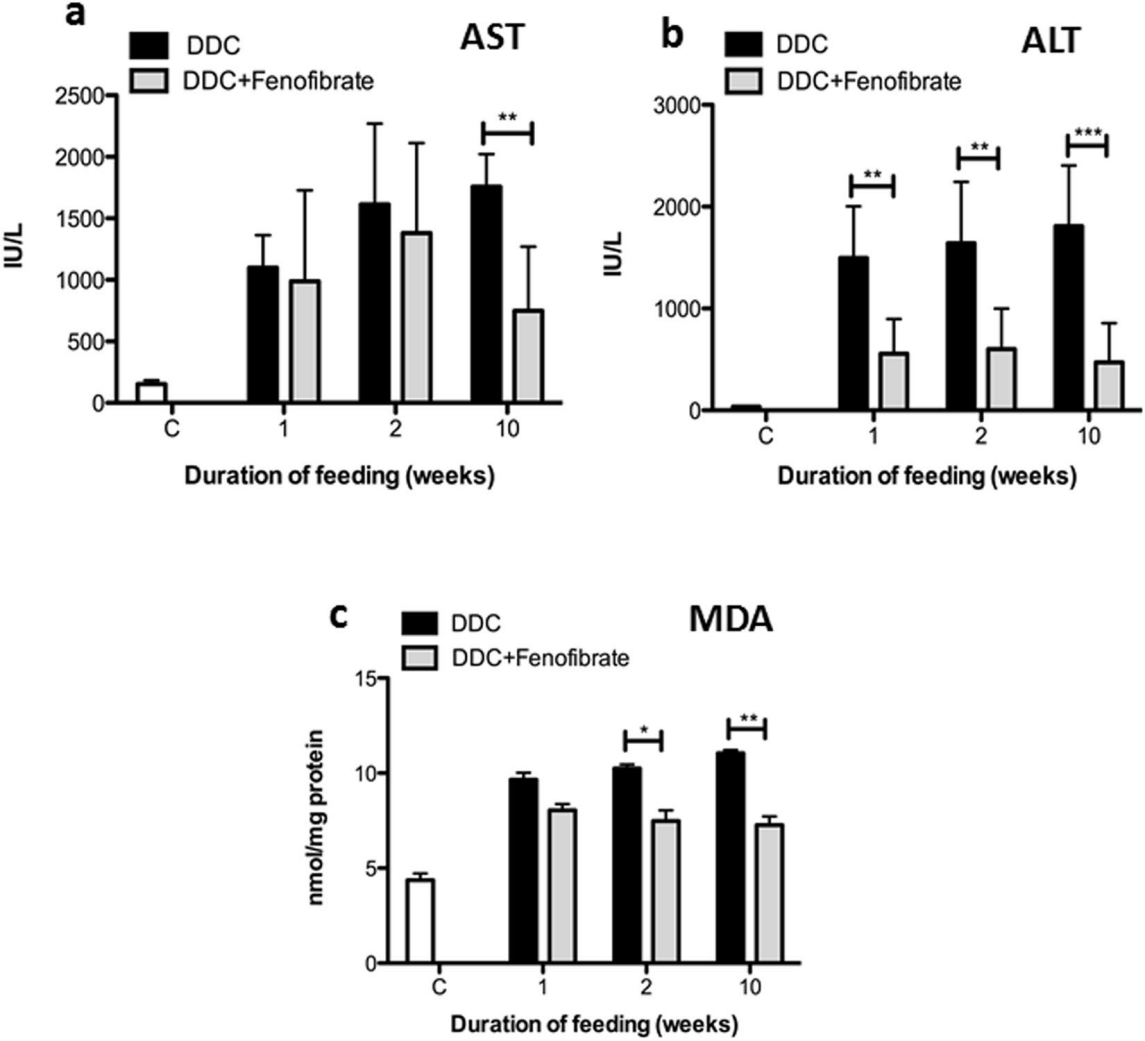
Fenofibrate prevents liver damage during DDC intoxication. Serum enzymes and liver tissue oxidative damage parameters. (**a**) Aspartic transaminase (AST); (**b**) alanine transaminase (ALT); (**c**) MDA-protein adduct levels (pmol/mg protein) in liver homogenates. Data are shown as mean ± SD collected from 5 animals per treatment group. Significances of DDC intoxication stages are shown versus DDC/fenofibrate co-administration: *p < 0.05; **p < 0.01; ***p < 0.001.

We found that DDC/fenofibrate co-treatment ameliorated overall hepatocellular damage, as indicated by lower activity of alanine aminotransferase (ALT), but did not restore it to control level (Fig. 3a). Interestingly, aspartate aminotransferase (AST) levels, reflecting mitochondrial damage, only improved in the group treated for 10 weeks (Fig. 3b). Moreover, oxidative damage after long-term DDC intoxication was significantly reduced by fenofibrate as indicated by significantly decreased formation of malondialdehyde (MDA) -protein adducts (Fig. 3c).

We checked the possibility that fenofibrate directly reduced the effectiveness of DDC by measuring the accumulation of protoporphyrin IX, and found no indications of reduced porphyrinogenic activity in DDC-fenofibrate co-treated mice (see Supplementary Fig. S2) compared to mice treated with DDC alone. In contrast, the accumulation of protoporphyrin IX was significantly higher in fenofibrate co-treated mice after 10 weeks (p < 0.001), for yet unknown reasons.

### Fenofibrate prevented DDC-mediated downregulation of PPARα-dependent genes involved in lipid metabolism

**Figure 4:**
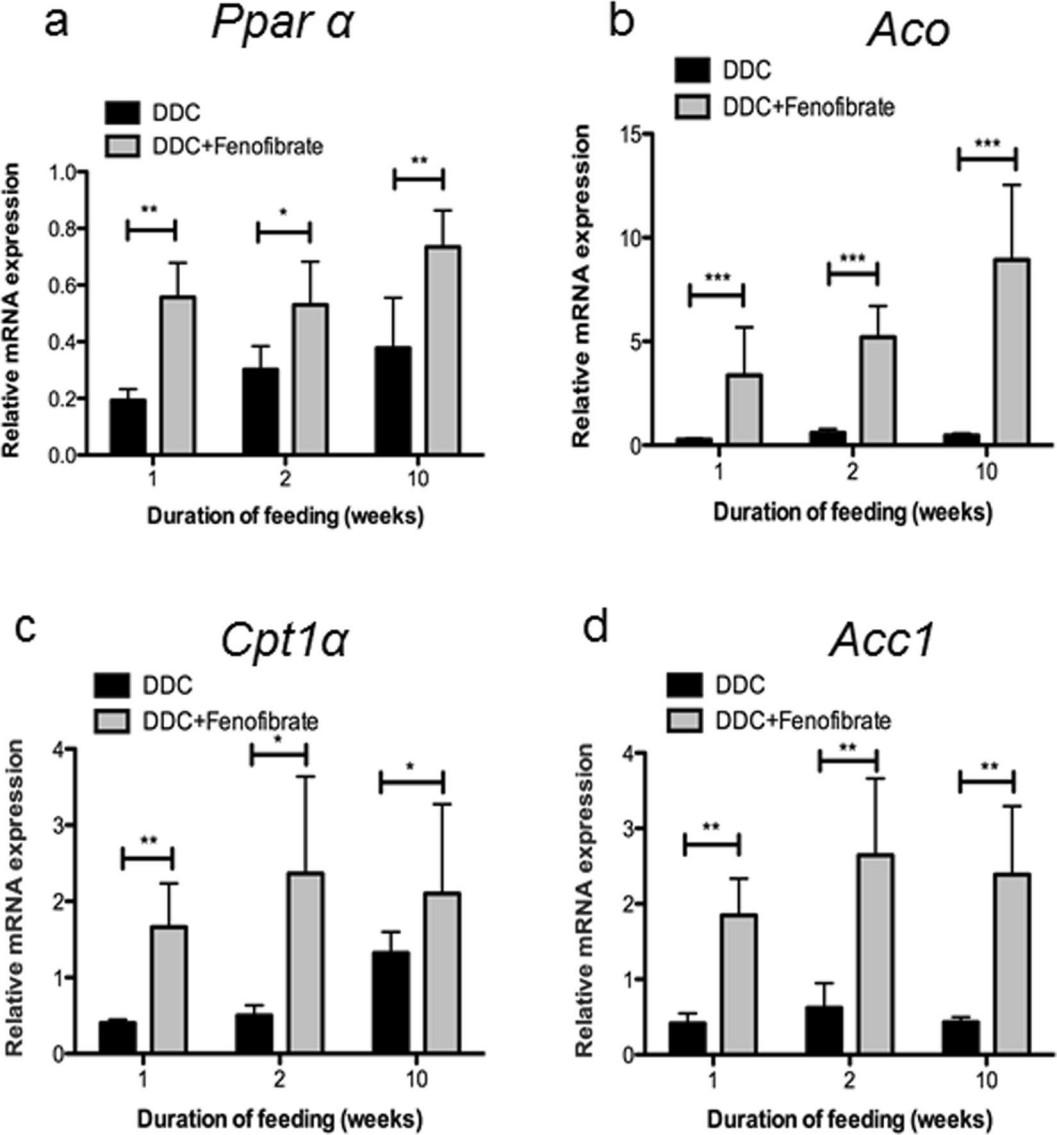
Effect of DDC/fenofibrate co-administration on expression levels of *Pparα* and lipid metabolism-related genes. (**a**) Normalized expression levels of *Pparα*; (**b**) acyl-CoA Oxidase (*Aco)*; (**c**) acyl-CoA carboxylase 1 (*Acc1)*; (**d**) carnitine-palmitoyl transferase 1α (*Cpt1α*) were evaluated by qRT-PCR of mRNA extracted from whole liver tissue. Data are given as fold-change relative to controls. Significances of DDC intoxication stages are shown versus DDC/fenofibrate co-administration: *p < 0.05; **p < 0.01; ***p < 0.001.

In line with the restored *Pparα* levels during DDC/fenofibrate co-treatment compared to intoxication with DDC alone (Fig. 4a) was the highly significant expression of PPARα-dependent *Aco*, *Cpt1α*, and *Acc1* genes (Fig. 4b–d; all data normalized to controls).

### Restoration of stress response by fenofibrate during DDC treatment

**Figure 5:**
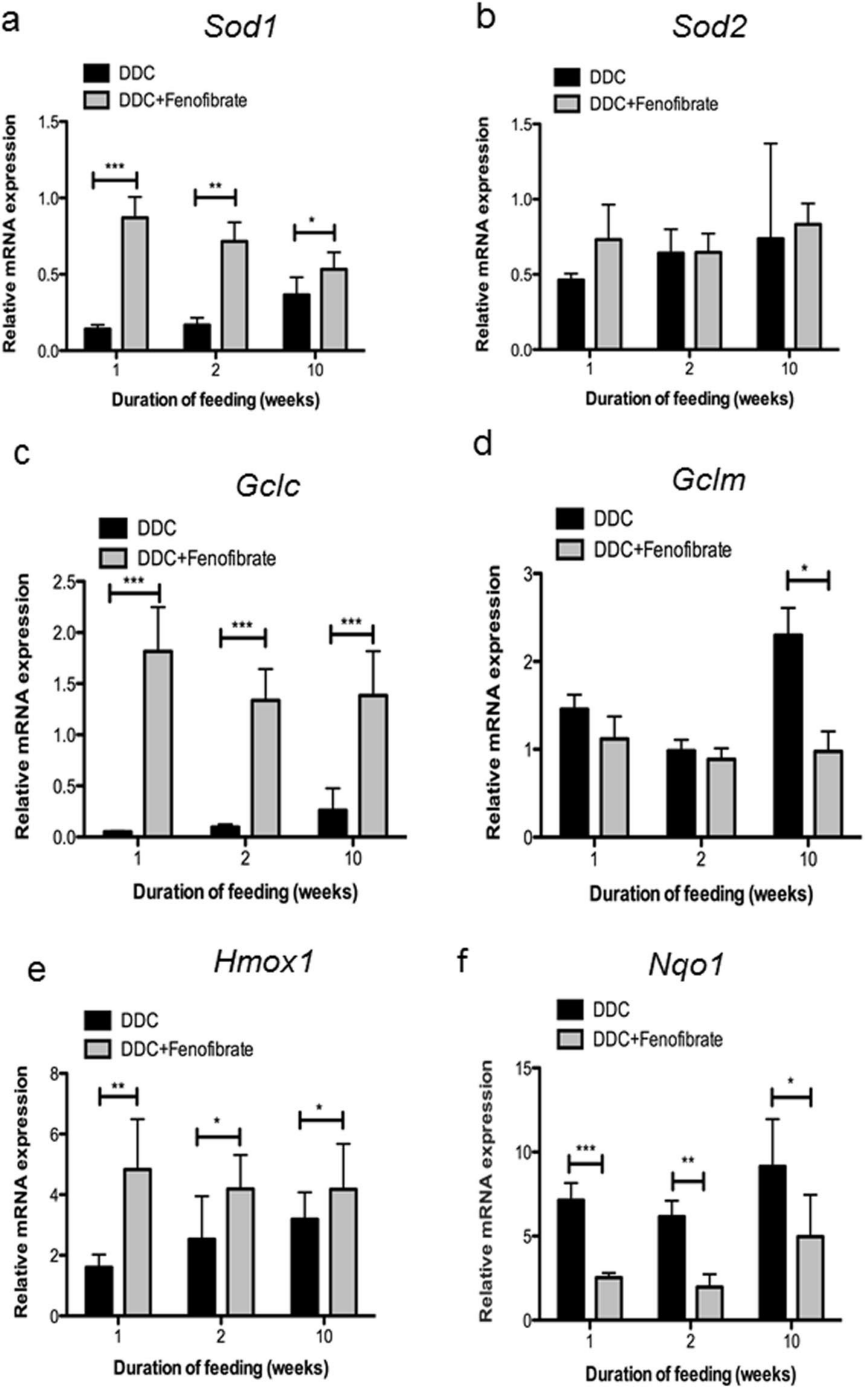
Effect of DDC/fenofibrate co-treatment on expression levels of antioxidant and stress–response genes. Normalized expression levels of (**a**) superoxide dismutase 1 (*Sod1)*, (**b**) superoxide dismutase 2 (*Sod2)*, (**c**) glutamate-cysteine ligase, catalytic subunit (*Gclc)*, (**d**) glutamate-cysteine ligase, modifier subunit (*Gclm)*, (**e**) heme oxygenase 1 (*Hmox1)*, and (**f**) NAD(P)H dehydrogenase quinone 1 (*Nqo1)* were evaluated by qRT-PCR of mRNA extracted from whole liver tissue. Data are given as fold-change relative to controls represented as mean ± SD collected from five animals per treatment group. Significances of DDC intoxication stages are shown versus co-administration of DDC and fenofibrate: *p < 0.05; **p < 0.01; ***p < 0.001.

Reactivation of PPARα by DDC/fenofibrate co-treatment led to increased expression of stress response genes. We found normalized expression of *Sod1, Sod2*, and *Gclc* (Fig. 5a–c). The twofold upregulation of *Gclm* expression after 10 weeks of DDC treatment (Fig. 5d) was reversed. Moreover, we observed enhanced induction of *Hmox1* (Fig. 5e) already after short-term intoxication. *Nqo1* expression was significantly downregulated by fenofibrate compared to DDC treatment alone, both at the short- and long-term intoxication stages (Fig. 5f; all data normalized to controls).

### Fenofibrate did not improve mitochondrial respiration

We had previously reported that during the first two weeks of DDC feeding mitochondrial function was severely impaired^11^. Mitochondrial respiration with succinate as respiration substrate was not improved by DDC/fenofibrate co-administration, and only insignificantly improved with NAD^+^-linked substrates (glutamate/malate) (see Supplementary Fig. SF3), which is in line with the observation that AST values remained unaltered.

### Reduction of inflammatory response by fenofibrate

**Figure 6:**
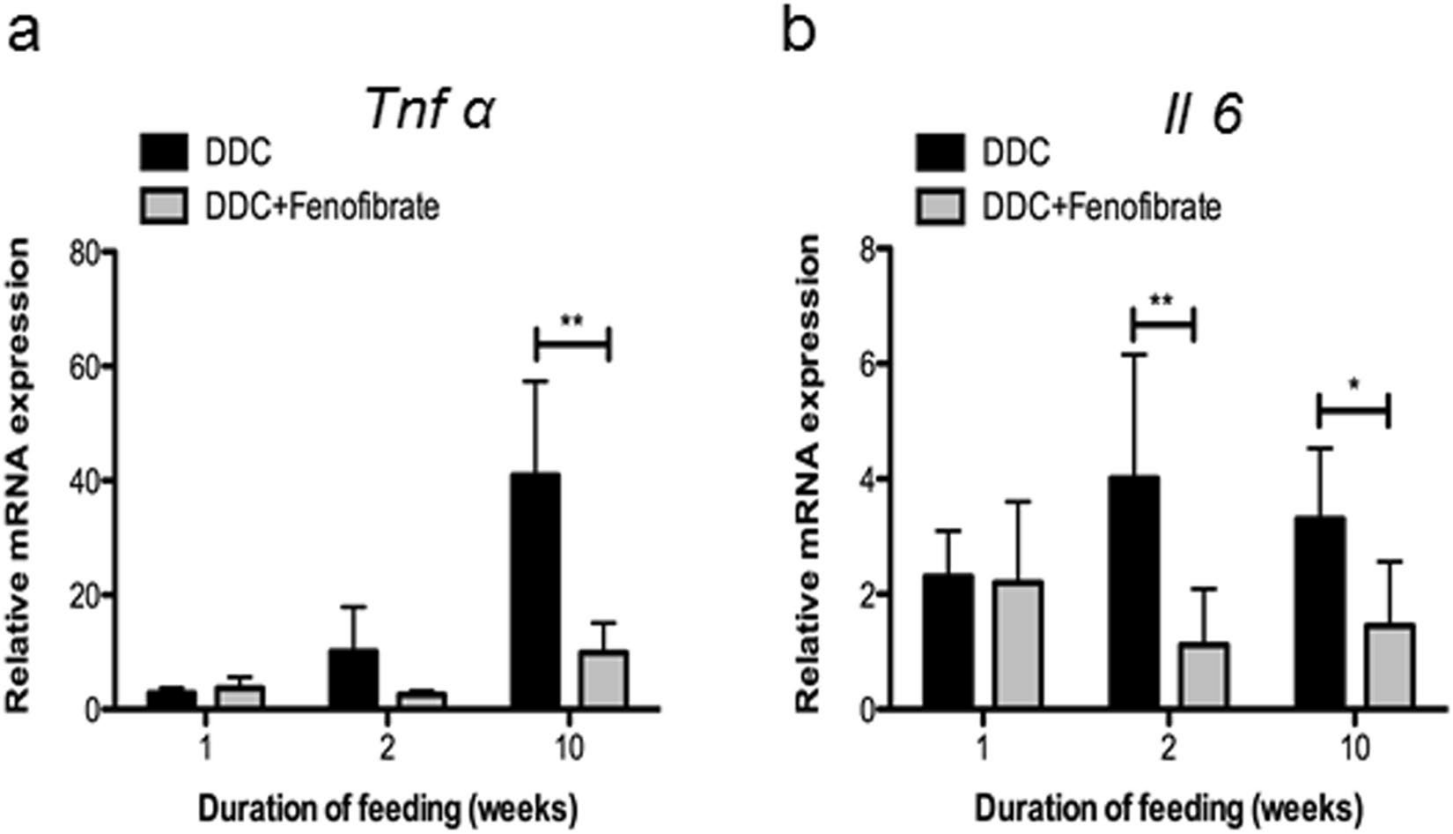
Fenofibrate reduces expression levels of inflammatory genes: (**a**) *Tnf*α; (**b**) *Il6*. Quantitative RT-PCR was performed with mRNA extracted from whole liver tissue. Data are given as fold-change relative to controls represented as mean ± SD collected from five animals per treatment group. Significances of DDC intoxication stages are shown versus co-administration of DDC and fenofibrate: *p < 0.05; **p < 0.01.

DDC intoxication for 2 and 10 weeks induced a strong inflammatory response as evident by the increased expression of the inflammatory cytokine genes *Tnfα* and *Il6* (Fig. 6a,b). Increased *TNF-α* expression was particularly striking in the long-term intoxicated group. DDC/fenofibrate co-administration significantly reduced the expression of these cytokines. To confirm these data we performed immunostaining for macrophages using F4/80 antibodies (see Supplementary Methods). Livers from mice treated with DDC for 10 weeks showed numerous F4/80-positive cells with predominance in lobular zones 2 and 3 (see Supplementary Fig. SF4), while their number was much lower in DDC-fenofibrate co-treated mice, largely resembling untreated controls.

### Effect of DDC/fenofibrate co-treatment on liver morphology in short- and long-term DDC-intoxicated mice

**Figure 7:**
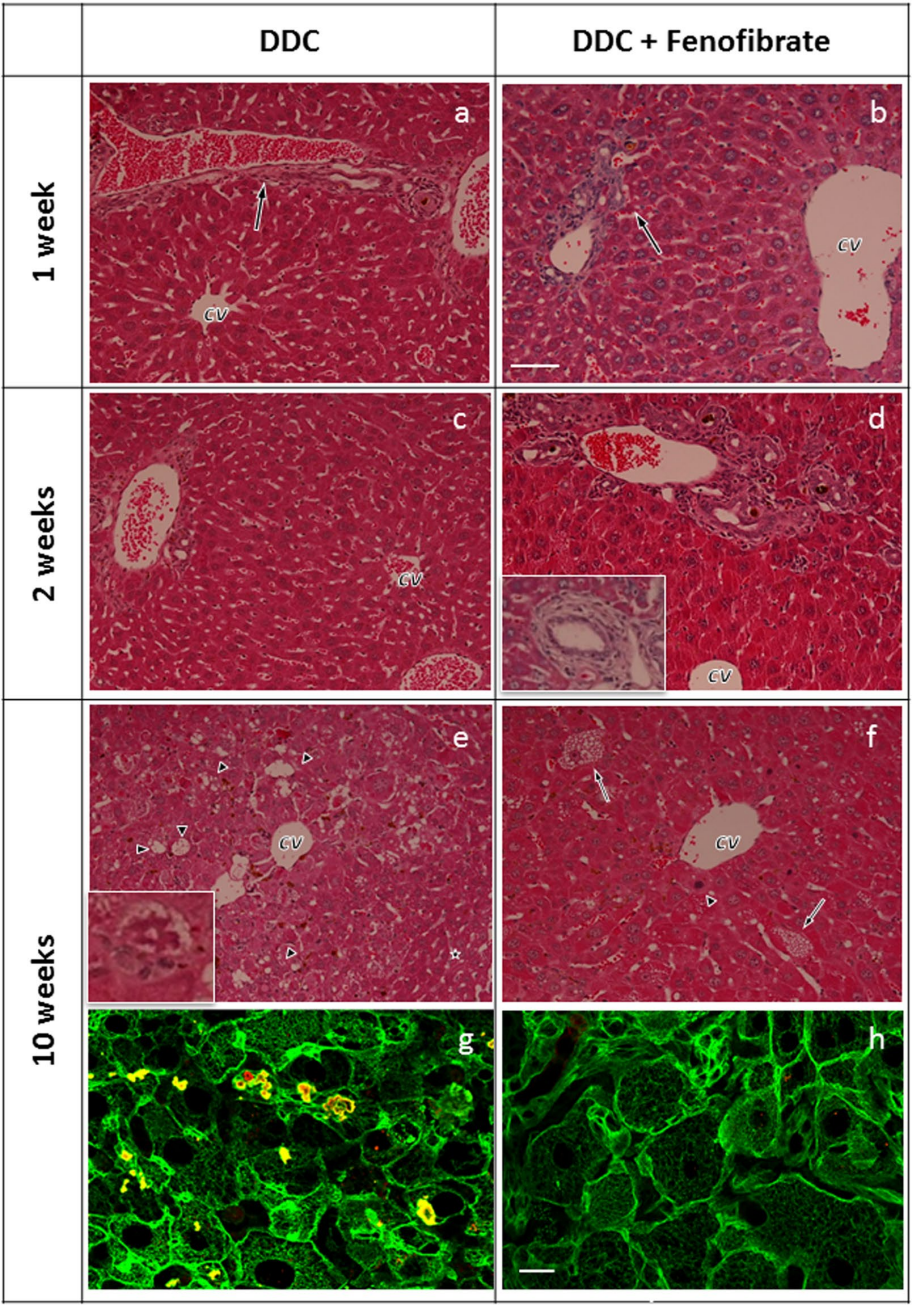
Liver morphology and immune fluorescence microscopy of DDC- and DDC/fenofibrate-treated mice. DDC treatment for 1 week with (**a**) or without (**b**) fenofibrate co-treatment results in irregular extension of portal tracts (arrows) with elongation of interlobular bile ducts displaying irregular epithelium mostly containing inspissated brown pigment plugs in their lumina. These features are exaggerated by DDC feeding for 2 weeks (**c**,**d**) but are identical in the DDC (**c**) and DDC/fenofibrate group (**d**), except for more eosinophilic homogenous cytoplasm in hepatocytes in the DDC/fenofibrate group; in some portal tracts bile ducts show signs of periductal sclerosis (arrow in the inset in (**d**)). DDC intoxication for 10 weeks (**e**) results (in addition to portal changes) in enlargement (ballooning) of centrilobular hepatocytes with enlarged nuclei, accumulation of fat vacuoles (arrowheads in (**e**); compare hepatocyte changes in the centrilobular area with smaller hepatocytes arranged in regular plates in the periphery of the lobule, asterisk) and formation of MDBs (inset in (**e**) with higher magnification). In contrast, co-treatment with fenofibrate (**f**) prevented MDB formation; hepatocytes in the centrilobular area are monomorphic and steatosis is much less pronounced (some hepatocytes with microvesicular fat accumulation are indicated by arrows; arrowhead denotes mitotic hepatocyte); (cv = central vein). Size bar is 40 µm. (**g**,**h**) Double immunofluorescence microscopy (red: p62; green: keratin 8/18). At 10 weeks of DDC treatment (**g**) we observe hepatocellular ballooning, loss, diminution or derangement of the keratin cytoskeleton and MDBs containing p62 and keratin (green + red → yellow). After DDC/fenofibrate co-administration for 10 weeks (**h**) hepatocytes appear focally enlarged, the keratin cytoskeleton is preserved, concentrated at the periphery of the hepatocytes, and no MDBs are seen. Size bar is 20 µm.

Hematoxylin and eosin (H&E) stained control liver is shown for comparison in Supplementary Fig. SF5. Livers of mice treated with DDC for 1 week showed slightly expanded portal tracts with increased numbers of bile duct profiles indicating tortuosity of interlobular bile ducts, a morphologic feature characteristic of mechanical interference with bile flow (Fig. 7a). They often contained dense plugs of brown pigment in their dilated lumina and were surrounded by few neutrophilic granulocytes. Pigment granules in the cytoplasm of hepatocytes as well as pigment-containing lobular and portal macrophages were sparse. Pigment deposition and portal changes were exaggerated after DDC intoxication for 2 weeks (Fig. 7c); moreover, a mild ductular reaction as well as accumulation of “oval cells” at the portal-parenchymal interface was observed. In addition, few necrotic hepatocytes were dispersed throughout the lobules. Apoptotic bodies and mitotic hepatocytes were rarely seen. DDC/fenofibrate feeding for 1 and 2 weeks resulted in almost identical portal morphology and pigment accumulation. However, in contrast to DDC feeding alone, hepatocytes were increased in size and displayed an eosinophilic, finely granular cytoplasm (Fig. 7b,d). Moreover, in about 50% of the animals fed for 2 weeks we observed variably sized areas of coagulative necrosis with marginal haemorrhage and focal neutrophil-granulocytic infiltration. This indicates that no strict correlation exists between serum biochemical parameters of liver damage and light microscopy.

DDC treatment for 10 weeks resulted in conspicuous expansion of portal tracts, fibrosis (including periductal fibrosis) and complete and incomplete portal-portal septa formation (Fig. 7e,f). Interlobular bile ducts were partly atrophic. Ductules extended into the lobules. Pigment (protoporphyrin IX) was abundant in hepatocytes, portal and lobular macrophages and bile duct lumina. The salient morphologic feature was enlargement (ballooning) of centrilobular hepatocytes, most of them containing small, medium-sized and large cytoplasmic fat vesicles together with classical MDBs. Lobular and portal infiltration by lymphoid inflammatory cells was mild to moderate. The number of F4/80-positive cells was increased (see Supplementary Fig. SF4). Surrounding neutrophils (satellitosis) were absent. Apoptotic bodies and mitotic hepatocytes were present at low numbers. DDC/fenofibrate co-treatment for 10 weeks had no influence on portal tract morphology and pigment content. However, in contrast to intoxication with DDC alone, enlarged hepatocytes predominantly in centrilobular position displayed eosinophilic, finely granular cytoplasm and only very mild steatosis (mostly below 5% of the parenchymal area) with small to medium-sized fat vacuoles, but lacked ballooning and MDB formation. The intermediate filament cytoskeleton was preserved as revealed by immunofluorescence microscopy (Fig. 7g,h). An immunofluorescence image of control liver is shown in Supplementary Fig. SF5, for comparison.

Steatohepatitic alterations in mice treated for 10 weeks with DDC or DDC/fenofibrate were graded and staged according to the proposal by Kleiner^24^ and an activity score was determined based on the intensity of steatosis, lobular inflammation (without taking into account the number of F4/80 positive cells) and hepatocellular ballooning in H&E stained sections (see Supplementary Table ST1). The activity scores were 6.0 +/−0.83 for 10 weeks DDC-treated and 0.83 +/−0.75 for DDC/fenofibrate co-treated mice. Fibrosis appeared less pronounced in the DDC/fenofibrate co-treated group, which was corroborated by image analysis after staining with PicroSirius red (10 weeks DDC: 12.51 +/−3.83%, 10 weeks DDC/fenofibrate: 5.96 +/−1.57%; see Supplementary Fig. SF6).

### Upregulation of the proteasomal component Psmd4, and reduced autophagy during DDC/fenofibrate co-treatment

**Figure 8:**
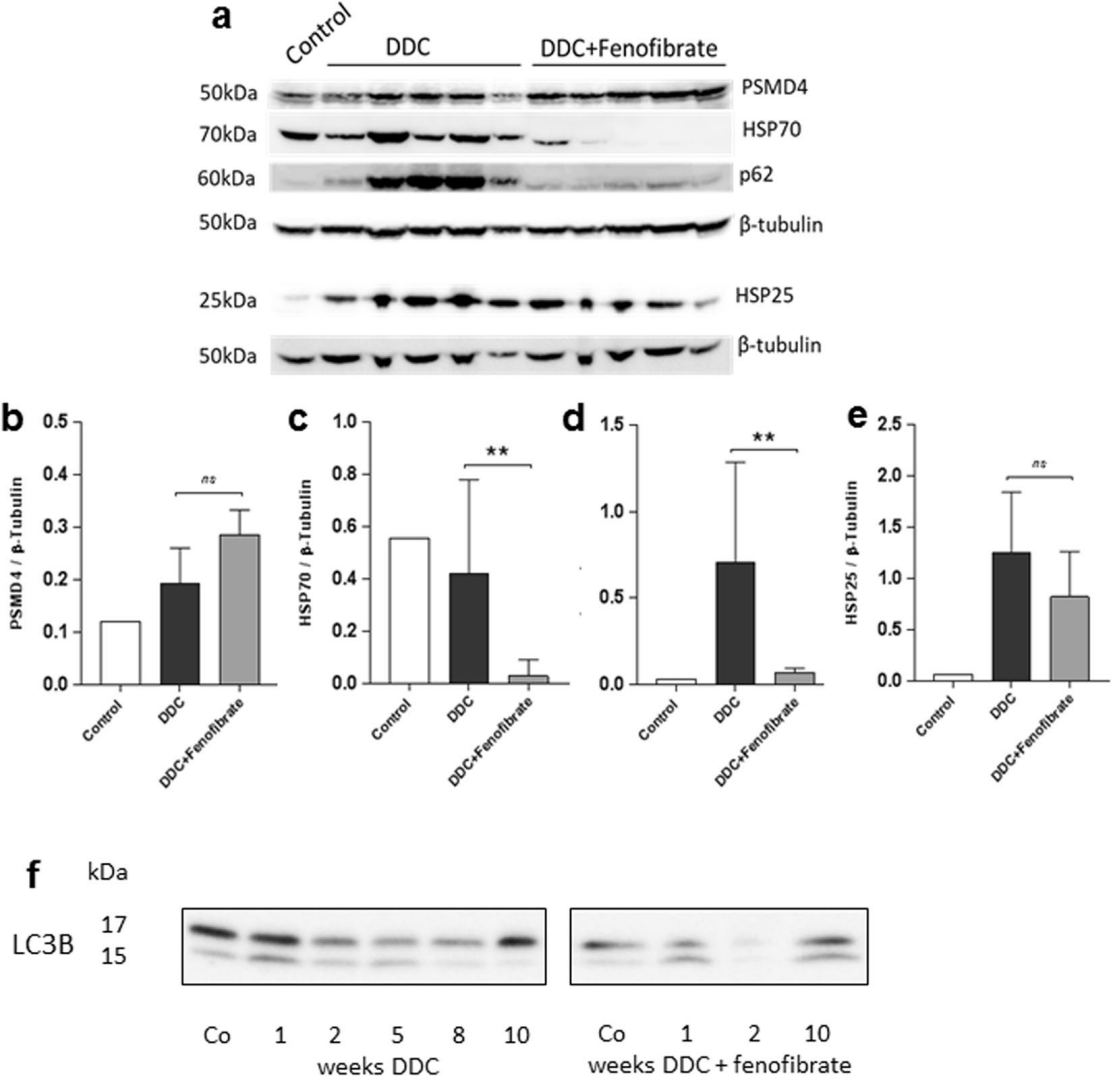
Western blot analysis of a proteasomal component (PSMD4), p62, Hsp25 and Hsp70, and of autophagy markers LC 3 I and LC 3 II (full size blots are shown in Supplementary Fig. SF8). (**a**) Western blots of liver homogenate from control mice (pool of n = 3), 10 weeks DDC treated mice (n = 5) and DDC/fenofibrate co-treated mice (n = 5). Densitometric analysis and quantification normalized to β-tubulin for PSMD4 (**b**), Hsp70 (**c**), p62 (**d**) and Hsp25 **(e**). Student’s t-test was performed to evaluate significance, *p < 0.05; **p < 0.01. Western blots of autophagy markers LC3 I and II in livers of mice treated with DDC (left) and DDC + fenofibrate (right) (full size blots are shown in Supplementary Fig. SF9).

It was previously described that activation of PPARs by a PPAR pan-agonist (WY-14643) increased important genes involved in proteome maintenance, such as chaperones and proteasomal proteins^25^. Hence, a possible effect of fenofibrate regarding the reduction of DDC-induced MDBs could be the increase of proteasomal activity, or the improved stabilization of partially misfolded protein by fenofibrate-mediated induction of chaperones. We therefore examined by Western blotting the presence of non-keratin key components of MDBs (p62, Hsp25, Hsp70)^26^, as well as a proteasomal component (Psmd4) in liver homogenates (Fig. 8) of chronically DDC-intoxicated mice, with and without fenofibrate treatment. Indeed, we found significant upregulation of Psmd4, indicating increased proteasomal content in DDC/fenofibrate co-treated livers. This is supported by immunofluorescence evaluation of ubiquitin, showing that ubiquitinated high molecular-weight protein (probably MDBs) was only observed in livers of mice DDC-intoxicated for 10 weeks, whereas after co-administration of fenofibrate, only few, small ubiquitinated aggregated were found, comparable to controls (see Supplementary Table ST2). Similarly, ubiquitinated high molecular-weight protein, shown by Western blotting, were elevated only in 10 weeks DDC-treated livers (see Supplementary Fig. SF7).

Hsp25 was not affected by fenofibrate, whereas p62 that was strongly overexpressed in DDC treated animals remained at control level in the DDC/fenofibrate co-treated group (Fig. 8). Hsp70 protein was strongly decreased in 10 weeks DDC/fenofibrate compared to DDC-only treated mouse livers. Likewise, Hsp70 mRNA expression showed a 10.2 +/−0.5fold increase over controls in mice treated for 10 weeks with DDC, which was reduced significantly by DDC/fenofibrate co-treatment (6.6-fold +/−0.6, compared to controls; p < 0.001).

In addition, we investigated autophagic activity (Fig. 8f) and observed that during DDC-treatment total LC3 levels dropped, suggesting increased degradation. Moreover, autophagic flux increased during DDC treatment as indicated by the increased LC3 II to LC3 I ratio (Fig. 8f, left panel). Co-treatment with DDC and fenofibrate showed reduced autophagic activity and flux (Fig. 8f, right panel).

## Discussion

Intoxication of mice with DDC caused specific metabolic alterations as well as a spectrum of histologic features in the liver, in particular disruption of the hepatocytic keratin intermediate filament cytoskeleton, ballooning of hepatocytes and formation of MDBs, equivalent to human steatohepatitis; the development of this phenotype was correlated with down-regulation of PPAR-α. Moreover, inflammatory markers, like elevated *Il-6* and *Tnfα* expression, observed in the later stages of DDC intoxication, were also found in patients with ASH underlining the clinical relevance of our model^27^. Co-treatment with fenofibrate, a potent PPARα agonist, reversed these changes: together with PPARα the levels of *Aco*, *Cpt1α* and *Acc1* involved in lipid metabolism were restored or even elevated. More importantly, fenofibrate enhanced the stress response by upregulating *Hmox1*, *Sod1*, and *Gclc*. Probably as a consequence, in the chronic intoxication stage, MDA-adducts and serum AST, reflecting oxidative stress and mitochondrial damage, were decreased in the DDC/fenofibrate-treated animals. It is particularly noteworthy, that fenofibrate treatment completely suppressed the morphologic features of steatohepatitis as well as inflammation as revealed by decreased *TNF-α* and *IL-6* expression and reduced macrophage infiltration.

Formation of cytoplasmic protein aggregates, like MDBs, depends on the balance between production of misfolded proteins and related rescue mechanisms, such as chaperone-dependent refolding, proteasomal degradation or autophagy^28^. We have previously shown that increased keratin synthesis and K8 > K18 ratio, as well as p62^29^, are essential for MDB formation in the DDC model^28^. Fenofibrate treatment moderately but significantly increased proteasomal Psmd4 compared to DDC treatment alone. Concomitantly, ubiquitin-containing protein aggregates that were present in 10 weeks DDC-treated livers were absent in DDC/fenofibrate co-treated livers, which may be attributed to enhanced proteasomal activity or decreased formation of aggregates. Whether the selective downregulation of Hsp70 has any relationship to the prevention of MDB formation is at present not clear, however, it is more likely that it is due to the overall normalization of metabolism, along with increased PPARα activity. P62 was upregulated at late stages of DDC-treatment and also in human steatohepatitis^30^ and is required for maturation and stabilization of MDBs^29^ and is thus not only an essential component of MDBs, but was found to activate the Nrf2-dependent antioxidant response^29,31,32^. However, since we found p62 at control levels in DDC/fenofibrate co-treated mice, the activation of the stress response under these conditions is probably independent of p62. It is noteworthy that fenofibrate led to normalization of a broad spectrum of steatohepatitic features. We think that the lower autophagic activity in DDC/fenofibrate co-treated livers is a consequence of largely reduced protein aggregation.

PPARα plays a prominent role in the development of human metabolic liver disease^4^ and its agonists have been suggested for prevention and treatment of steatohepatitis (reviewed in^7^). Based on the findings presented here we consider the downregulation of PPARα with its consequences a key event in steatohepatitis. The fact that PPARα-deficient mice develop steatosis rather than steatohepatitis with MDBs^33,34^ suggests that the appearance of the typical steatohepatitis phenotype requires an additional “hit” that most likely targets the suppressed stress response. The cause of PPARα deficiency both in humans and disease models is unclear and warrants further detailed studies. Two mechanisms may be proposed: (i) downregulation by ligands of the aryl hydrocarbon receptor (AhR)^35^. In DDC-treated liver, AhR ligands are endogenously produced either in the course of oxidative stress (oxidation products of polyunsaturated fatty acids or amino acids, like tryptophan^36^) or from bilirubin and other heme derivatives^37^ as a consequence of porphyria, and increased degradation of heme resulting from upregulation and mitochondrial translocation of HO-1^11^; (ii) stabilization of hypoxia-inducible factors by DDC-induced oxidative stress^38,39^. Since the PPARα promoter contains a hypoxia-responsive element that is present both in the human and mouse genes^38^ we postulated previously that the mitochondrial dysfunction ensuing from DDC intoxication leads to a pseudohypoxic state^11^ responsible for permanent downregulation of PPARα.

PPARα and related downstream genes were decreased immediately after onset of DDC treatment. Whereas *PPARα*, *Aco*, and *Acc1* remained suppressed during the whole experimental period, *Cpt1α* expression rose again after the initial drop, and so did the stress response genes *Sod1*, *Gclc* and *Hmox1*. Normally, toxic insults rapidly induce stress response genes via Nrf2 activation. In contrast, in our experimental setting, prolonged DDC treatment was required to increase expression of the ‘typical’ stress response genes, like *Hmox1* or *Gclm*. Interestingly, also mitochondrial function improved somewhat in the chronic intoxication state concomitant with the appearance of the steatohepatitis phenotype; this suggests that the characteristic morphology represents an adaptive cell response^11^. Cross-talk between PPARα and the stress response pathways governed by Nrf2 was previously shown to affect a broad range of target genes^40–42^. Particularly *Hmox1* has been reported to depend on the nuclear translocation of Nrf2 under oxidative stress^43,44^.

In a previous study we showed that DDC treatment of mice adversely affected hepatic mitochondrial function and related energy production and led to early oxidative damage^11^. Mitochondrial dysfunction as revealed by decreased mitochondrial respiration rate and hepatic ATP content became most evident two weeks after commencement of intoxication. Delayed stress response, therefore, coincided with mitochondrial damage and contributed to increased lipid peroxidation. It is, therefore, likely that mitochondrial dysfunction with ATP depletion exacerbates DDC toxicity. This is in line with observations in a variety of human disorders including steatohepatitis (ASH, NASH) and drug-induced liver disease in which ATP depletion is also a prominent feature^45^.

We conclude that the DDC-induced rapid downregulation of PPARα in the liver is responsible for impaired and delayed antioxidant/stress response and lipid metabolism. Both can be restored by the PPARα agonist fenofibrate, indicating, that the observed effects are intimately related to PPARα. Furthermore, fenofibrate prevented the formation of the morphologic key features of steatohepatitis, namely MDBs, ballooning, and disruption of the keratin cytoskeleton. PPARα downregulation and related impairment of fatty acid metabolism and blunted stress response may act as the *priming* event that sensitizes the liver to the consequences of mitochondrial dysfunction^11^. Since our model in its metabolic and morphologic aspects closely resembles the human disease, activation of PPARα has potential as causal therapeutic option: even though fenofibrate has shown adverse effects in the treatment of fatty liver disease^7^ other PPARα agonists presently in clinical trials may prove better suited for therapy of NASH^8^.

## Material and Methods

All chemicals were of analytical grade or better, and provided by Sigma-Aldrich, Vienna, Austria, unless noted otherwise.

### Animals and diets

Male Swiss Albino mice (strain Him OF-1; Institute of Laboratory Animal Research, Medical University of Vienna, Himberg, Austria), aged 8–12 weeks, were used in all experiments and kept at a 12-hour light/dark cycle with *ad libitum* access to food and water. The animal experiments were approved by the Austrian Federal Ministry of Science, Research and Economy, Division of Genetic Engineering and Animal Experiments (Vienna, Austria) (BMWFW-66.010/0113-WF/II/3b/2014) and were performed in accordance with the relevant legal regulations and ethical guidelines.

After acclimatization on control diet in our animal facility for at least 2 weeks (all diets were obtained from Ssniff Spezialdiäten GmbH, Soest, Germany) mice were randomly assigned to different experimental groups (n = 5 per group). Mice received control diet supplemented with 0.1% DDC for 1, 2, 5, 8, and 10 weeks, respectively (1 and 2 weeks represent the acute stage, 5 weeks the intermediate, and 8 and 10 weeks the chronic intoxication stage). The effect of fenofibrate was assessed by feeding mice control diet supplemented with both 0.1% DDC (Sigma-Aldrich, Vienna, Austria) and 0.2% fenofibrate (Sigma-Aldrich). The combined DDC/fenofibrate feeding was restricted to the 1, 2 and 10 weeks stages.

Food consumption and weights of the animals were monitored weekly as previously described with emphasis on estimation of food wasting.

At the end of the different feeding periods, mice were sacrificed by cervical dislocation after overnight fasting and the livers were removed. Blood was collected by heart puncture.

### Histology

Sections (3 μm thick) of formaldehyde-fixed paraffin-embedded liver tissues were deparaffinised and stained with hematoxylin and eosin (H&E) following standard procedures. They were light-microscopically evaluated by a liver pathologist (H. D.).

### Immunofluorescence microscopy

Double-label immunofluorescence microscopy was performed for the evaluation of the keratin intermediate filament cytoskeleton and MDB formation on cryosections of liver tissue stored in liquid nitrogen as described previously^22,46^. Primary antibodies to p62 (polyclonal guinea pig antibody against p62; Progen, Heidelberg, Germany) and a monoclonal mouse anti-human/mouse keratin K8 (IgG1, clone Ks 8.7, Progen) were used. Alexa Fluor 488 nm-conjugated goat anti-mouse IgG (Molecular Probes, Leiden, Netherlands), t-methylrhodamine-isothiocyanate-conjugated pig anti-rabbit Ig (Dako, Glostrup, Denmark), Rhodamine Red-X-conjugated goat anti-guinea pig IgG (Jackson Laboratories, Germany), and FITC-conjugated rabbit anti-mouse IgG (Zymed, San Francisco, CA, USA) were used as secondary antibodies. Specimens were analyzed with a Zeiss LSM 510 laser-scanning confocal microscope (Zeiss, Oberkochen, Germany).

### Serum parameters of liver damage

Serum aspartate transaminase (AST), and alanine transaminase (ALT) activities were measured using a Hitachi 917 analyzer (Boehringer Mannheim, Germany) and commercially available assays at the diagnostic laboratory of the pediatric clinic of the Medical University of Graz, Austria.

### MDA-protein adduct assay

Lipid peroxidation was assessed by quantification of malondialdehyde (MDA)-protein adducts (MDA-protein-adduct ELISA Kit, Cell Biolabs Inc., San Diego, U.S.A.) according to the manufacturer’s protocol. Briefly, BSA standards and/or protein samples (10 μg/mL) were allowed to adsorb for 2 hours at 37 °C on a 96 well plate. MDA-protein adducts were detected using an anti-MDA antibody, followed by a horseradish peroxidase-conjugated secondary antibody. For quantification, an authentic MDA-bovine serum albumin standard (Cell Biolabs) was used. This procedure has the advantage over the conventional MDA/thiobarbituric acid-reactive substances assay that lipid peroxidation is assessed as it had occurred *in vivo*.

### Quantitative reverse transcriptase polymerase chain reaction analysis (qRT-PCR)

Total hepatic RNA was isolated using TRIzol reagent (Invitrogen, Vienna, Austria) and reverse transcribed into cDNA (Invitrogen) according to the manufacturer’s protocol. qRT-PCR was performed on a 7900 T Fast Real Time PCR system (Applied Biosystems, Foster City, USA) using SYBR Green as the detection fluorophore. Primer sequences are reported in Supplementary Table ST3. Data are shown as expression ratios of target genes normalized to the expression of *Gapdh* as internal reference in each sample. Quantitative RT-PCR data were analyzed by the 2^−ΔΔCt^ method^47^.

### Western Blot Analysis

Whole tissue extracts were prepared using radio immunoprecipitation assay (RIPA) buffer (Thermo Scientific, Vienna, Austria). Protein concentration was quantified with the Bradford assay (Pierce Coomassie Plus (Bradford) Assay Reagent, Bio-Rad). Equal amounts of proteins (40 µg/lane) were separated using Nu-PAGE Bis-Tris 4–12% Gels (Life Technologies, Vienna, Austria) and transferred to a PVDF membrane after blocking with 5% blotting grade skimmed milk (Bio-Rad). Detection of blotted proteins was performed using antibodies PSDM4/S5a (Cell Signalling Technology, Danvers MA, USA; CST #3846 S, dilution 1:400), HSP25 (Abcam, Cambridge, UK, ab202846; 1:1000), p62 (Progen, GP62C, 1:1000) and HSP70 (Santa Cruz Biotechnology, Heidelberg, Germany; sc-024; 1:1000), normalized to β-tubulin (1:1000; Cell Signalling Technology, #2128 S). The bound antibodies were visualized with a horseradish-peroxidase-conjugated secondary antibody (P0260, P0217, P0141; DakoCytomation, Dako), using Pierce ECL Western Blotting Substrate (Thermo Scientific) on an ImageQuant LAS 500 gel imaging system (GE Healthcare, Vienna, Austria). Densitometric quantification was performed using Image lab software.

For detecting the relative levels of LC3B-I and LC3B-II, protein homogenates from indicated stages of DDC were were resolved on Nu-PAGE Bis-Tris 4–12% Gels (Life Technologies, Vienna, Austria) and transferred to a PVDF membrane after blocking with 5% blotting grade skimmed milk (Bio-Rad). Membranes were then probed with the primary antibody against microtubule-associated protein light chain 3 (LC3B) (#2775, Cell Signaling Technology, Danvers MA) at a 1:1000 dilution. HRP-conjugated goat anti-rabbit (1:2500) secondary antibodies (Dako, Glostrup, Denmark) were used and chemiluminescence was detected in response to SuperSignal™ West Pico (Thermo Scientific) on a ChemiDoc™ MP imaging system (Bio-Rad Laboratories). The signal of relative LC3II against LC3I was used to interpret the level of ongoing autophagy.

### Statistical analysis

Statistical analysis was performed using GraphPad Prism (San Diego, USA). Data are shown as mean +/− standard deviation. For the time course study, data were compared by one-way ANOVA followed by Bonferroni’s multiple-comparison test for various groups. DDC/fenofibrate group data were compared to the respective DDC-fed groups by student’s t-test. P < 0.05 was considered significant. All data generated or analysed during this study are included in this published article.

## Electronic supplementary material

Supplementary Information

## Data Availability

The manuscript, figures and supplement show all data available for this study.
