# Supplementary material for: The PPARα Agonist Fenofibrate Prevents Formation of Protein Aggregates (Mallory-Denk bodies) in a Murine Model of Steatohepatitis-like Hepatotoxicity
Source: Sci Rep. 2018 Aug 28;8:12964. doi: 10.1038/s41598-018-31389-3 (PMC6113278; doi:10.1038/s41598-018-31389-3)
Supplement: Supplementary file 1 — Supplementary Information [file 41598_2018_31389_MOESM1_ESM.pdf]

Supplementary Data for

The PPARα Agonist Fenofibrate Prevents Formation of Protein Aggregates (Mallory-Denk bodies) in a Murine Model of Steatohepatitis-like Hepatotoxicity

Aniket Nikam[1,3], Jay V. Patankar[2,4], Meghana Somlapura[1], Pooja Lahiri[1], Vinay Sachdev[2], Dagmar Kratky[2], Helmut Denk[1], Kurt Zatloukal[1], and Peter M. Abuja[*1]

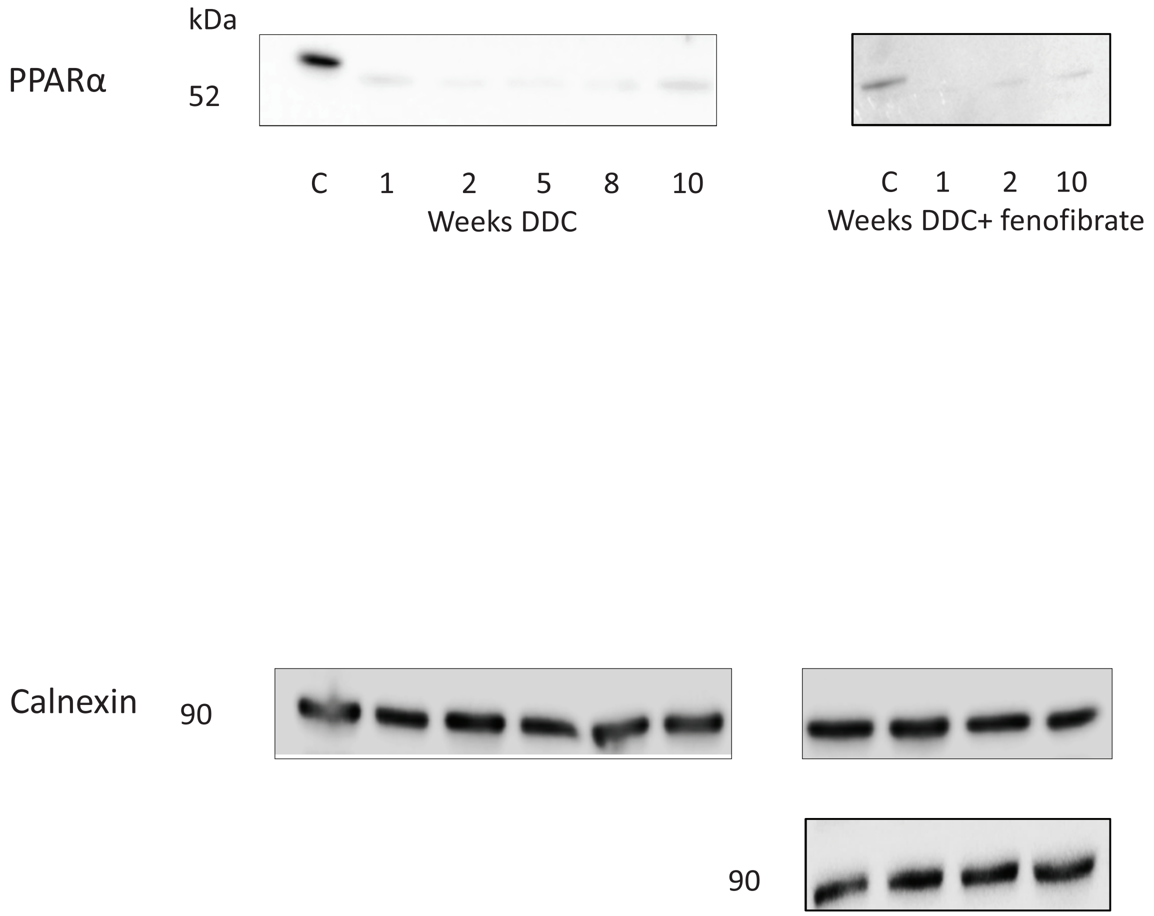

Supplementary figure SF1: Western blots of PPARα of liver homogenates from mice treated with DDC and DDC + fenofibrate, respectively. Calnexin was used as a loading control. Full size blots are shown in Supplementary Figure SF9

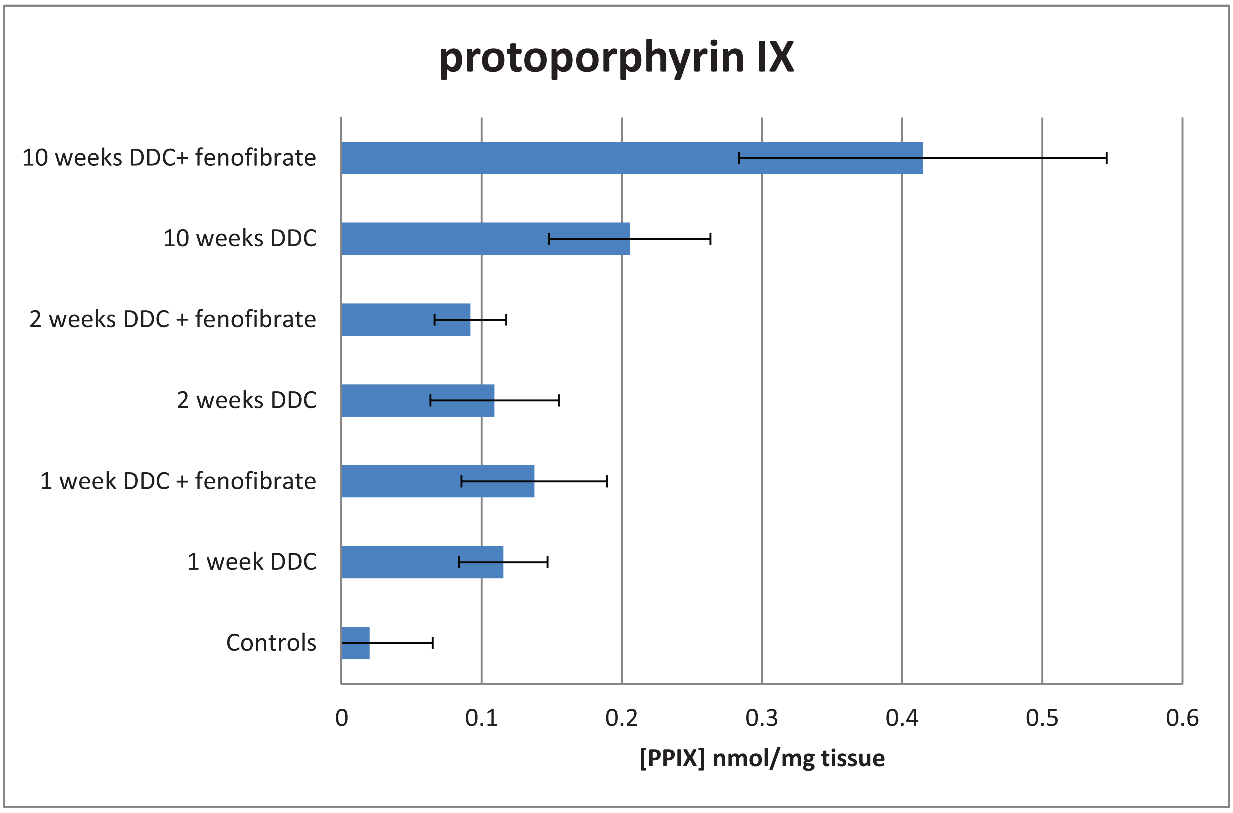

Supplementary figure SF2: Analysis of tissue protoporphyrin IX (PPIX). PPIX was extracted from cryopreserved mouse liver of the indicated experimental groups and analysed as described in Supplementary Methods. Statistical significance was assessed by 1-way ANOVA: 10 weeks DDC/fenofibrate was significantly different from all other groups ($p < 0.001$), 10 weeks DDC was significantly different from controls ($p < 0.01$).

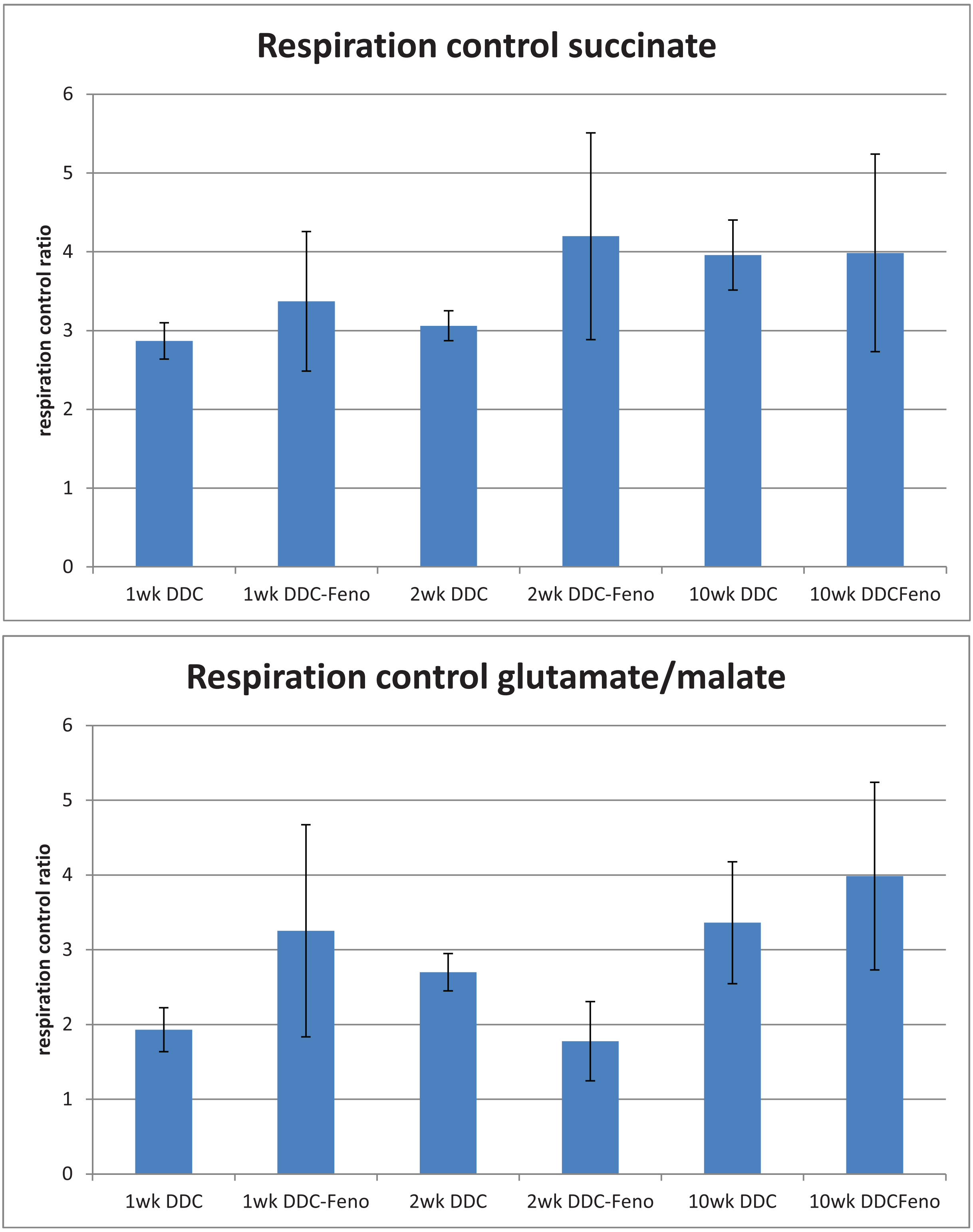

Supplementary figure SF3: Mitochondrial respiration control ratios – respiration with substrate (5 mM succinate; top panel, 2.5 mM each glutamate/malate, bottom panel) in presence vs. absence of 250 µM ADP is shown. Significance was assessed by 1-way ANOVA with Bonferroni correction.

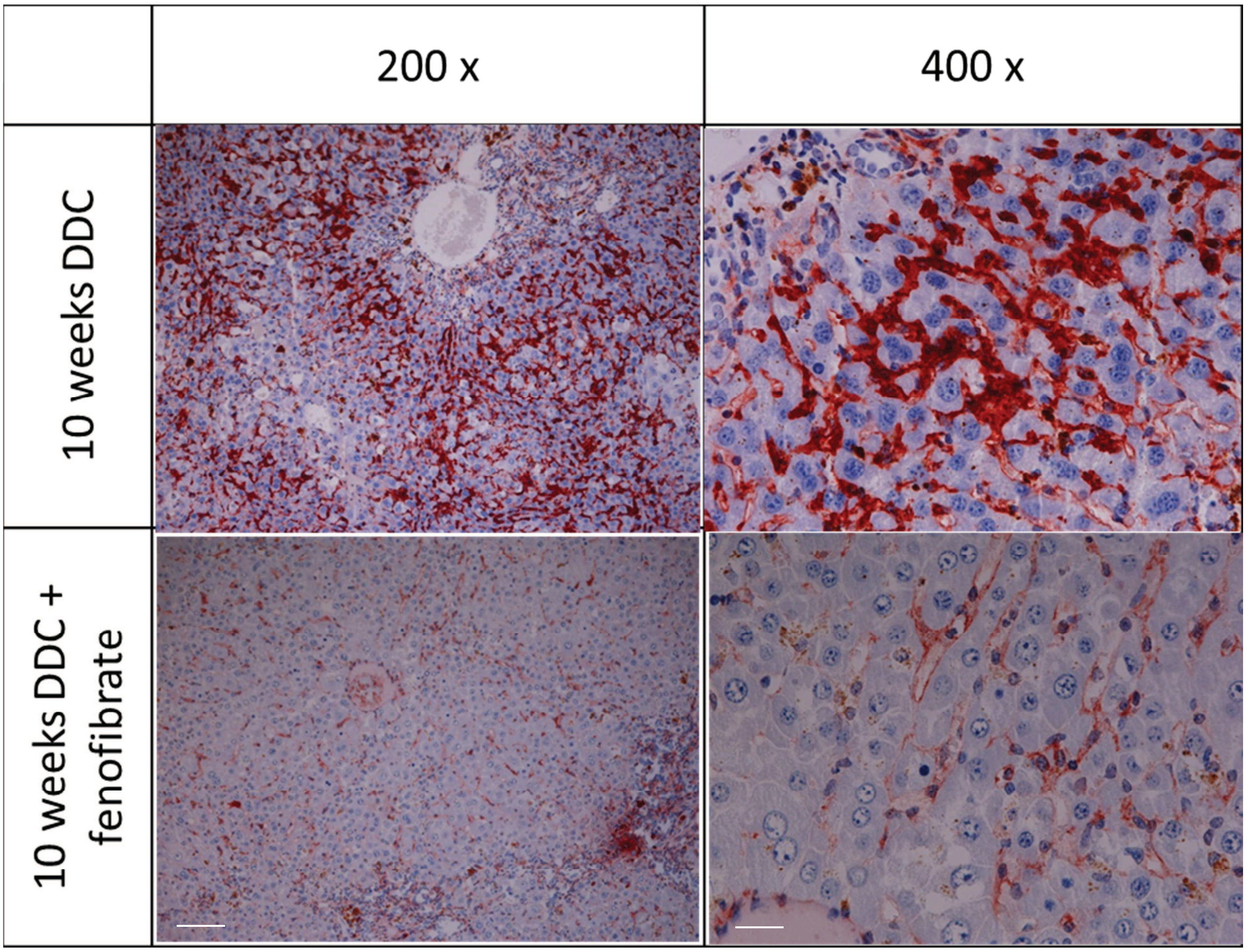

Experimental group	F4/80 positive cells	Remarks
**Controls**	moderate infiltration	centrilobular, sometimes diffuse
**10 weeks DDC**	moderate – strong infiltration	diffuse or centrilobular
**10 weeks DDC + fenofibrate**	little – moderate infiltration	diffuse or portal

Supplementary Figure SF4: Immunostaining and scoring for macrophages (F4/80 antibody). Representative images of formalin-fixed sections of mouse liver (top: after 10 weeks DDC-treatment, bottom: 10 weeks DDC + fenofibrate treatment) were stained against F4/80 antigen (red staining). Size bar = 40 µm (200x), 20 µm (400x).

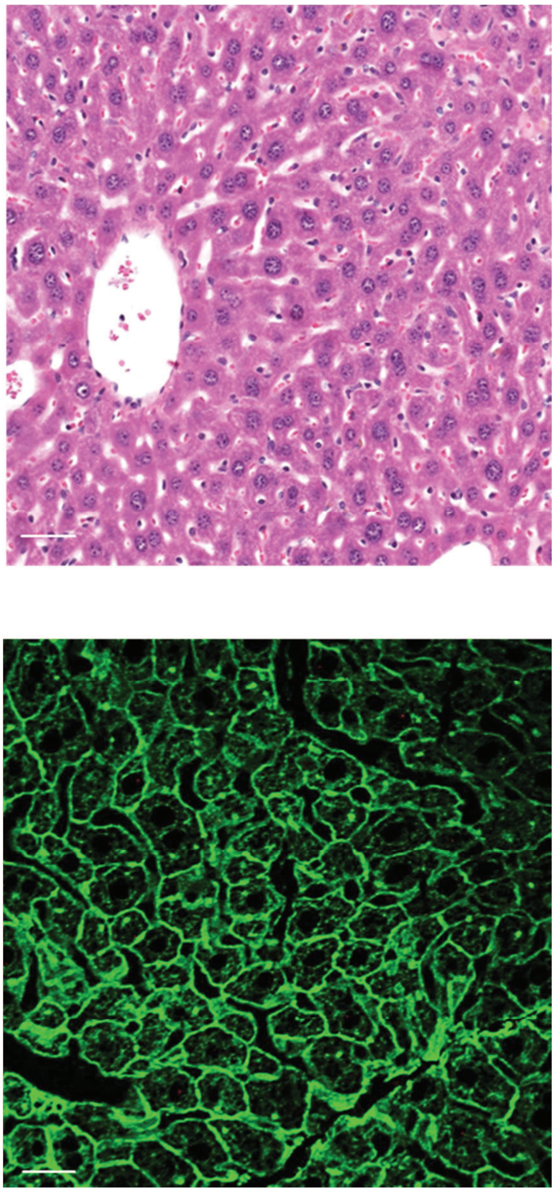

Supplementary Figure SF5: Morphology of control liver, and immunofluorescence microscopy

Top: formalin-fixed section stained with hematoxylin and eosin (bar = 40 µm). Bottom: cryosection stained as described in Methods. Red fluorescence: p62, green fluorescence: keratin 8/18. (bar = 20 µm)

Sample	animal	Steatosis[1] grade	Lobular inflammation[2] grade	Ballooning[3] grade	NAFLD[4] activity score (NAS)	MDB score	Portal inflammation[5] score	Fibrosis[6] stage
10 weeks DDC	1	2	2	3	7	Prominent	2	3
	2	2	1	3	6	Prominent	1	3
	3	1	2	3	6	Prominent	1	3
	4	1	2	2	5	Prominent	1	3
10 weeks DDC + fenofibrate	1	0	1	0	1	None	1	2
	2	0	1	0	1	None	1	2
	3	1	1	0	2	None	2	2
	4	0	0	0	0	None	1	2
	5	1	0	0	1	None	1	3
	6	0	0	0	0	None	1	3

Supplementary Table ST1: NAS scoring of 10 week DDC mouse livers with and without fenofibrate co-treatment. H&E stained were scored according to Kleiner[24] at a magnification of 200x. Average NAS score for 10 weeks DDC was 6.0 +/- 0.82, for DDC/fenofibrate co-treatment 0.83 +/- 0.75.

[1]Steatosis (0: none; 1: < 33%; 2: 33% - 66%; 3: > 66%)
[2]Lobular inflammation (0: none; 1: 1-2 foci; 2: 2-4 foci; 3: > 4 foci)
[3]Ballooning (0: none; 1: mild; 2: prominent; 3: marked)
[4]NAFLD activity score (NAS): (grades of) steatosis + ballooning + lobular inflammation
[5]Portal inflammation (0: none; 1: mild; 2: moderate; 3: severe)
[6]as proposed by Kleiner[24]

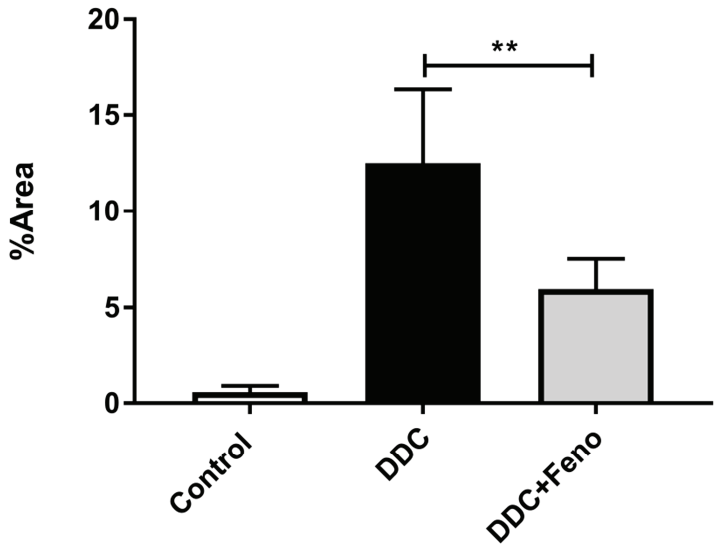

Supplementary Figure SF6: Quantification of collagen.

Formalin-fixed tissue sections from control mice, 10 weeks DDC treated mice and DDC+Fenofibrate co-treated mice (n=5) were stained with PicroSirius red and quantified using ImageJ. Data are represented as mean ± SD. Student's t-test was performed to evaluate significance, *: $p < 0.05$; **: $p < 0.01$

Stage	DDC	DDC + Fenofibrate
Controls	none	---
1 week DDC	Very few cells contain 1-2 positive granula	Very few cells contain 1-2 positive granula
2 weeks DDC	Few cells contain positive granula, few larger aggregates	Very few cells contain 1-2 positive granula
10 weeks DDC	Many large positive aggregates, centrilobular, partly peripheral	Very few cells contain 1-2 positive granula

Supplementary Table ST2: Ubiquitin immunofluorescence staining and scoring. Cryosections of liver were stained according to the protocol given in Supplementary Methods and scored by an experienced pathologist (H.D.).

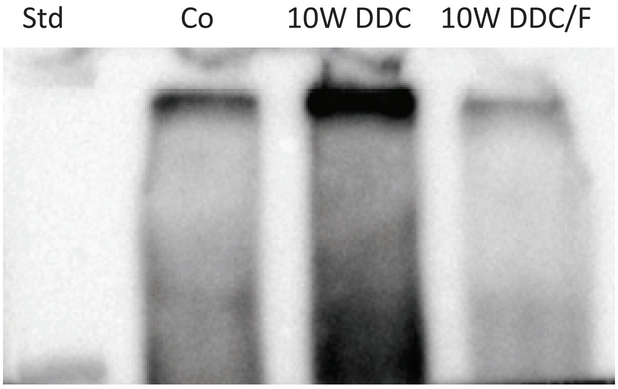

Supplementary Figure SF7: High molecular-weight ubiquitin-containing aggregates. Pooled homogenates of mouse liver (controls, 10 weeks DDC, 10 weeks DDC/fenofibrate) were separated for western blotting. The stacking gel area was blotted separately and incubated with anti-mouse ubiquitin antibody to visualise high molecular mass ubiquitin-protein aggregates.

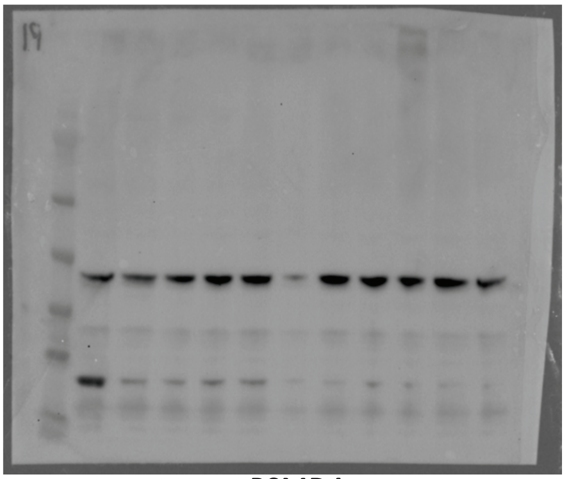

PSMD4

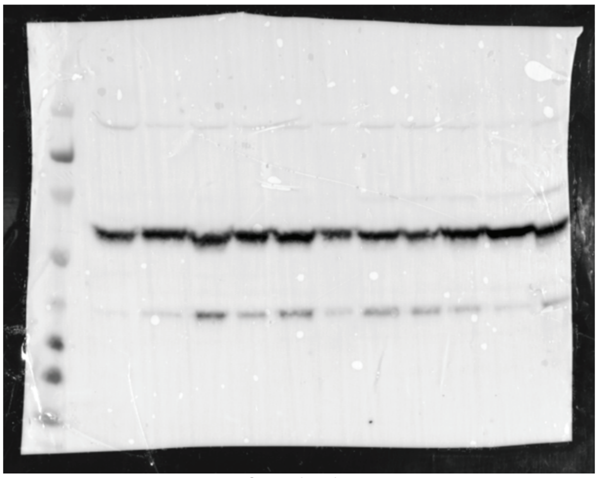

β-tubulin

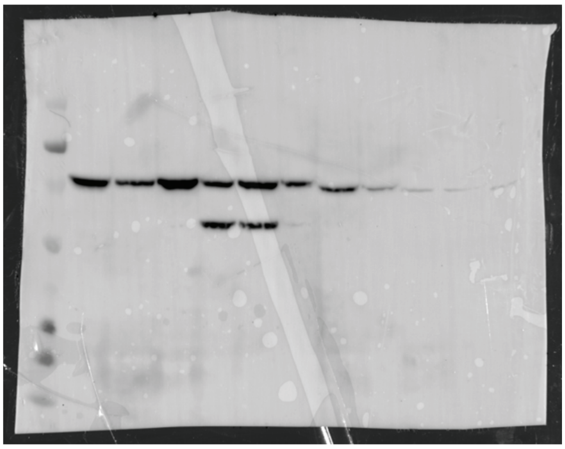

Hsp70

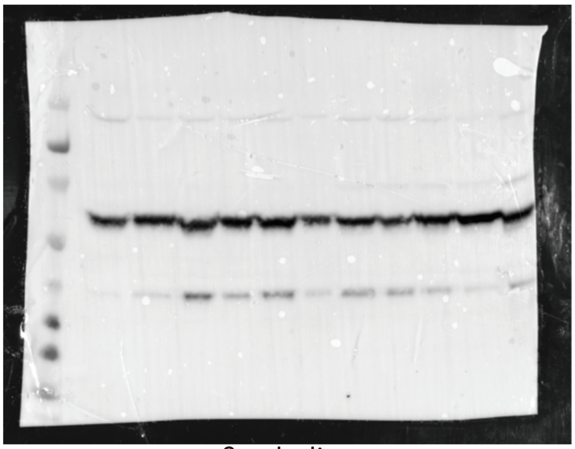

β-tubulin

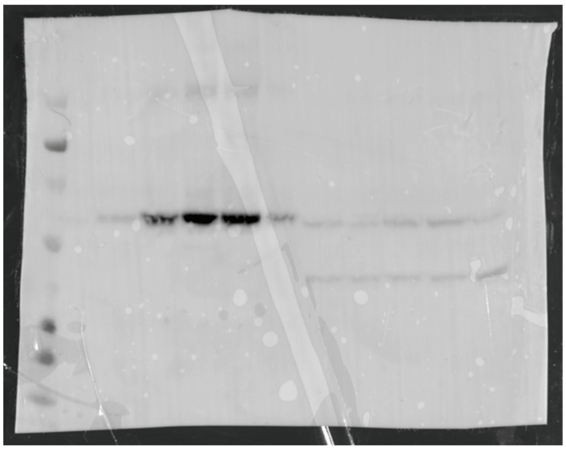

p62

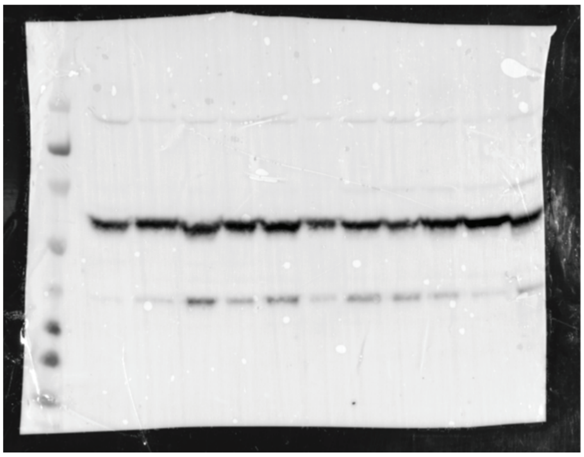

β-tubulin

Supplementary Figure SF8 (1st part): Full size blots to Figure 8a: PSMD4 and β-tubulin run on separate gels using same amount of protein. Hsp70, p62 and β-tubulin blotted from same gel. Continued with 2nd part. First lane is marker, other lanes are as in Figure 8a.

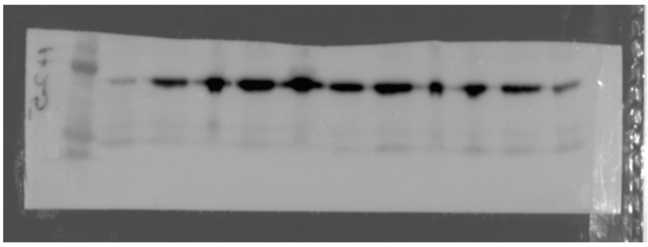

Hsp25

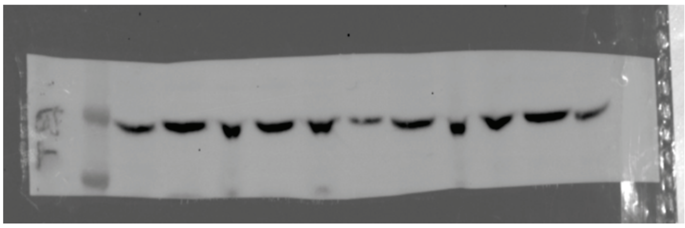

β-tubulin

Supplementary Figure SF8 (2nd part): Full size blots to Figure 8a: Hsp25 and β-tubulin were run on the same gel, the blot was cut to separate molecular weights. First lane is marker, other lanes are as in Figure 8a.

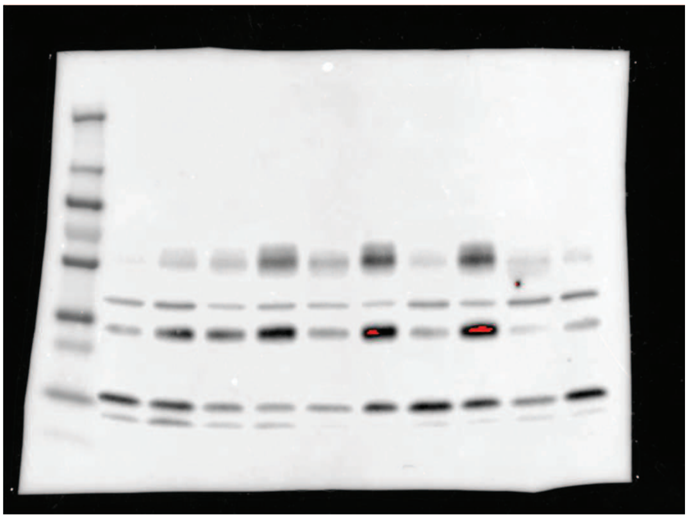

DDC

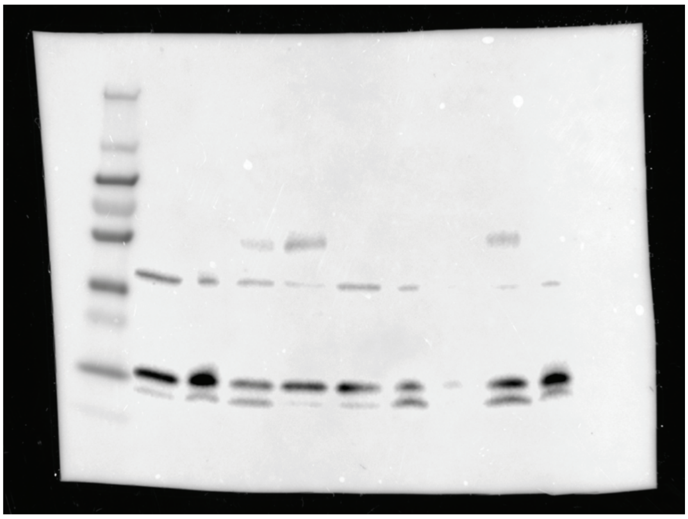

DDC + fenofibrate

Supplementary **Figure SF9: Full size blots to Figure 8f.** DDC: lanes left → right are marker, control, 1 week, 2 weeks, 5 weeks, 8 weeks, 10 weeks (control, 10 weeks, recovery, reintoxication not used in this work). DDC/fenofibrate: marker (control, 1 weeks DDC, 2 weeks DDC, 10 weeks DDC, repetition); control, 1 week DDC/fenofobrate, 2 weeks DDC/fenofibrate, 10 weeks DDC/fenofibrate (extra lane recovery, not used in this work).

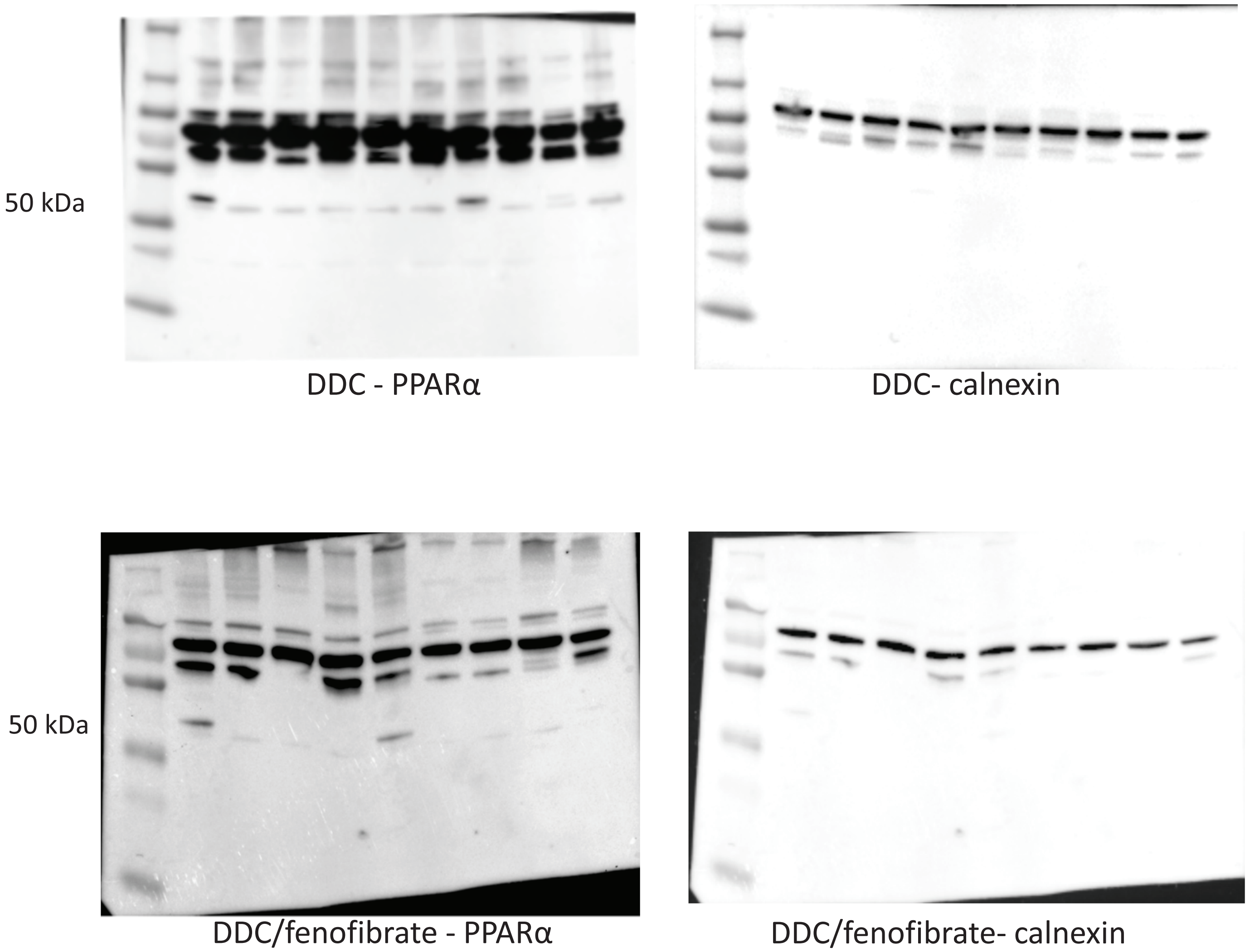

Supplementary Figure SF10: Full size blots to Supplementary Figure SF1: DDC: lanes left → right are marker, control, 1 week, 2 weeks, 5 weeks, 8 weeks 10 weeks. (Other lanes - controls, 10 weeks, recovery, reintoxication are not used in this work). DDC/fenofibrate: lanes left → right are marker (control, 1 week DDC, 2 weeks DDC, 10 weeks DDC, repetition); control, 1 week DDC/fenofibrate, 2 weeks DDC/fenofibrate, 10 weeks DDC/fenofibrate (last lane DDC/fenofibrate recovery not used in this work)

Supplementary Table ST3: Primer sequences used in the qRT-PCR experiments.

Gene	Forward primer sequence	Reverse primer sequence
PPARα	5' TCATCACAGACACCCTCTC 3'	5' ATATTCGACACTCGATGTTCAG 3'
Aco	5’ CCTGTTGGCCTCAATTACTC 3’	5’ GGTCATATGTGGCAGTGGTT 3’
Cpt1α	5' AGCGACTCTTCAATACTTCCC 3'	5' TGTTGGATGGTGTCTGTCTC 3'
Acc1	5'TAACCCAACTCAGCAAGACC 3'	5'GAAGCAATAAGAACCTGACGAG 3'
Sod1	5' CAATACACAAGGCTGTACCA 3'	5' CCACCATGTTTCTTAGAGTGAG 3'
Sod2	5' TGAGAAGTTTAAGGAGAAGCTG 3'	5' GGAATAAGGCCTGTTGTTCC 3'
Gclc	5' CATCATCAATGGGAAGGAAGG 3'	5' CATTAGTTCTCCAGATGCTCTC 3'
Gclm	5' GACCCGAAAGAACTGCTC 3'	5' AGCCTTTAGACTTGATGATTCC 3'
Hmox1	5' ACATTGAGCTGTTTGAGGAG 3'	5' GGTCTTTGTGTTCCTCTGTC 3'
Nqo1	5' GACCTTGCTTTCTATCACCA 3'	5' GATGCCACTCTGAATCGG 3’
Tnfα	5' CTCACACTCAGATCATCTTCTC 3'	5' CTTGGTGGTTTGCTACGA 3'
IL 6	5' TCTCTGCAAGAGACTTCCA 3'	5' CCTCCGACTTGTGAAGTG 3'

Supplementary methods

Preparation of mouse liver mitochondria

Animals (4-6 per group) were fasted overnight and sacrificed by cervical dislocation around 9.00 a.m.. Livers were excised, rinsed and minced in ice-cold mitochondrial isolation medium (10 mM HEPES, pH 7.4, 250 mM sucrose, 1 mM EDTA) to remove blood and bile. All following steps were performed on ice or at 4°C. Livers were homogenized in a Potter-Elvehjem homogenizer with a loosely fitting pestle in mitochondrial isolation medium containing 1 mg/ml fatty acid-free bovine serum albumin and 1 mM dithiothreitol (freshly added, to prevent accidental oxidation of a sensitive cysteine residue on complex I). The homogenate was centrifuged at 800 x g for 10 min in a Sorvall RC-5B high-speed centrifuge using a Sorvall SS-34 rotor (DuPont Instruments, Inula, Vienna, Austria) to remove nuclei and cell debris. The supernatant was centrifuged for 15 min at 6000 x g to pellet mitochondria. The pellet was washed once by re-suspending in 20 ml isolation medium (without EDTA), re-pelleted and re-suspended in little isolation medium to give an approximate protein concentration of 70 mg/mL. Protein content was determined by the Lowry method using the Bio-Rad protein reagent kit (Bio-Rad, Vienna, Austria).

Determination of respiratory control coefficient

Mitochondrial respiration was measured using a CLARK electrode (Oxygraph, Anton Paar, Graz, Austria) by incubating 1mg/ml of mitochondrial protein at 37°C in respiration buffer (70 mM sucrose, 220 mM mannitol, 2mM HEPES, 0.1% fatty acid-free bovine serum albumin, 1mM EDTA, 2.5 mM KH_2PO_4, 3 mM $MgCl_2$, pH 7.4), and 5mM of succinate as substrate, in presence or absence of 250 µM ADP. Alternatively, glutamate and malate (2.5 mM each) were used as NAD-linked substrates. Respiration control ratio (RCR) = [state 3 respiration rate (with ADP)] / [state 4 respiration rate (without ADP)].

Determination of mouse liver tissue protoporphyrin IX (PPIX)

For determination of PPIX we adapted an albumin-enhanced fluorescence method [1] to allow determination of PPIX without interference by bilirubin, extracting tissue by a slightly modified FOLCH procedure [2]. Briefly, frozen tissue (20 mg) was homogenized in a reaction tube in a total volume of 200 µL potassium phosphate buffer (KP_i; 100 mM, pH = 7.4), followed by 500 µL of methanol and 100 µL 5% trichloroacetic acid in H_2O. Tissue was homogenized with an immersion blender and then 800 µL $CHCl_3$ are added. All steps were performed on ice. The mixture was vigorously shaken for at least one minute, repeated two

times with an interval of 5-10 minutes on ice. Subsequently, phase separation was accelerated by centrifugation (100 x g, 1 min, 4°C). The lower phase containing PPIX was collected and dried in vacuum. The dry residue was dissolved in Tris-HCl-albumin buffer (100 mM, pH = 9.3, 300 μM bovine serum albumin) and fluorescence measured at 410/630 nm (ex/em) in a black microplate. For quantification, a standard series of authentic PPIX was used (0 .. 10 μM PPIX).

Quantitative RT-PCR

Total hepatic RNA was isolated using TRIzol reagent (Thermo Fisher Scientific, Germany) and DNase digestion plus RNA Cleanup were performed with the RNeasy MinElute Cleanup Kit (QIAGEN GmbH, Hilden, Germany) according to the manufacturer's protocol.

RNA was analyzed by reverse transcription into cDNA using the High-Capacity cDNA Reverse Transcription Kit (Thermo Fisher Scientific, Germany) according to the manufacturer's protocol. As primer and reaction mix we used (Bio-Rad, Vienna, Austria).

qRT-PCR was performed on a QuantStudio(TM) 7 Flex System (Applied Biosystems, Foster City, USA) using Power SYBR Green PCR Master Mix (Thermo Fisher Scientific, Wilmington, DE) as the detection fluorophore.

Western blotting

Western blotting was performed as given in the main text.

For detecting the relative levels of LC3B-I and LC3B-II, protein homogenates from indicated stages of DDC were separated on Nu-PAGE Bis-Tris 4-12% Gels (Life Technologies, Vienna, Austria) and transferred to a PVDF membrane after blocking with 5% blotting grade skimmed milk (Bio-Rad). Membranes were then probed with the primary antibody against microtubule-associated protein light chain 3 (LC3B) (#2775, Cell Signaling Technology, Danvers MA) at 1:1000 dilution. HRP-conjugated goat anti-rabbit (1:2500) secondary antibodies (Dako, Glostrup, Denmark) were used.

Chemiluminescence was detected by SuperSignal™ West Pico (Thermo Scientific) on a ChemiDoc™ MP imaging system (Bio-Rad Laboratories). The signal of relative LC3II against LC3I was used to interpret the level of ongoing autophagy.

Immunostaining of macrophages (F4/80)

After deparaffinizing, sections were treated with Protease Type XXIV (Sigma) 0,1% for 10 minutes, after washing (all washing steps performed with phosphate-buffered saline, pH = 7.2), blocked with 1% H_2O_2 for 10 minutes with Dako Blocking Solution, and then washed again. Antibody F4/80 (Serotec MCA 497 GA) 1:500 diluted in S2022 (Dako diluent) was then incubated for 60 min, followed by polyclonal rabbit-anti-rat Immunglobulins/HRP for 30 minutes and K5007 (DAKO) Envision+, also 30 minutes. As chromogen, AEC Substrate Chromogen ready to use (Dako) was incubated for 10 minutes. Counterstaining was Mayer`s haematoxylin for 30 seconds, followed by washing with tap water.

Staining with PicroSirius red (Collagen)

PicroSirius Red staining was performed to evaluate liver collagen. For quantification of the stained sections, the ImageJ software was used. The sections were scanned and a threshold defined by a certain intensity of staining was applied. The numerical value corresponding to the area of stained tissue above threshold represents the percentage of collagen deposition within the area.

References:

1 Athar, H., Ahmad, N., Tayyab, S. & Qasim, M. A. Use of fluorescence enhancement technique to study bilirubin-albumin interaction. *Int J Biol Macromol* **25**, 353-358, doi:S0141813099000562 (1999).

2 Folch, J., Lees, M. & Sloane Stanley, G. H. A simple method for the isolation and purification of total lipides from animal tissues. *J Biol Chem* **226**, 497-509 (1957).
